# Chlorin Conjugates in Photodynamic Chemotherapy for Triple-Negative Breast Cancer

**DOI:** 10.3390/ph17050576

**Published:** 2024-04-30

**Authors:** Meden F. Isaac-Lam

**Affiliations:** Department of Chemistry and Physics, Purdue University Northwest, Westville, IN 46391, USA; isaaclam@pnw.edu or isaaclam@purdue.edu

**Keywords:** photodynamic therapy, anti-cancer treatment, chemotherapy, photosensitizers, drug combinations

## Abstract

Breast cancer (BC) is the most common type of cancer in women and the number of new cases in the US is still increasing each year. Triple-negative breast cancer (TNBC), which comprises 15–20% of all breast cancer, is a heterogeneous disease and is considered the most aggressive type of breast cancer due to the lack of estrogen receptor (ER), progesterone receptor (PR), and human epidermal growth factor receptor 2 (HER2) expressions for treatments. Traditional chemotherapy is the standard protocol for the treatment of TNBC. Toxicity and multidrug resistance are major drawbacks to chemotherapy. The lack of molecular targets and poor prognosis for TNBC prompts an urgent need to discover novel therapeutic strategies to improve clinical outcomes and quality of life for patients. Photodynamic therapy (PDT) or light treatment is a binary anti-cancer procedure that uses a photosensitizer (PS) that, upon light activation, produces cytotoxic oxygen species, destroying tumor cells. PDT is minimally invasive and can be repeated a few times without accumulating significant toxicity in the surrounding tissues. The primary goal of this study was to investigate in vitro photodynamic chemotherapy as a ternary combination therapy using our synthesized photosensitizers (chlorin–vitamin conjugates and their corresponding indium complexes) co-treated with known chemotherapeutic agents (taxol, doxorubicin, cisplatin, fluorouracil, or methotrexate) in the presence of light and determine the optimum conditions as a pre-clinical study of an enhanced tumoricidal effect against TNBC. Our results indicated that the best combination for an effective chemophotodynamic effect involves a ternary treatment of the indium complex of the chlorin–lipoic acid conjugate (InCLA) co-treated with taxol, which exhibited strong synergism at the nanomolar concentration when combined in the presence of visible light irradiation. Other ternary combinations containing taxol with a synergistic anti-tumor effect against TNBC include chlorin–pantothenic acid (CPA) and chlorin–biotin (CBTN) conjugates. Several other ternary combinations containing InCLA, CBTN, and CPA with either cisplatin, fluorouracil, or methotrexate were identified to generate a synergistic or additive effect. The light dosage remained constant, but the dosages of photosensitizers and chemotherapy drugs were varied to obtain the lowest possible concentration for the desired effect. The synergistic, additive or antagonistic effects of the drug combinations were determined based on the Chou–Talalay method, with InCLA–taxol having the lowest combination index (CI) of 0.25. Fluorescence and transmission electron microscopy (TEM) images provided evidence of apoptosis as the preferred mode of cell death. Our study demonstrated the combination of PDT and chemotherapy as a potential treatment option for TNBC patients.

## 1. Introduction

Breast cancer (BC) is the most common type of malignancy in women. In 2024, there were 313,510 estimated new BC cases in the US [1]. BC, which is curable in about 75% of patients with early-stage non-metastatic cases, is a heterogeneous disease with molecular features that include the activation of growth factors and hormone receptors, and/or *BRCA* mutations [2,3]. Aside from local treatments such as surgery and radiation therapy, hormone therapy, targeted therapy, or chemotherapy are the conventional strategies used to treat BC patients. About 25% of patients in the early stages will progress to the metastatic stage, with a survival rate of 2–3 years depending on the BC subtype [4].

Triple-negative breast cancer (TNBC), which comprises 15–20% of all breast cancer, is considered the most aggressive type of breast cancer due to the lack of estrogen receptor (ER), progesterone receptor (PR), and human epidermal growth factor receptor 2 (HER2) expressions for treatments [5,6,7]. Patients with HER(+) receptor status have the most favorable prognosis, with the cancer growing more slowly, followed by the HER(−) subtype, and lastly, the TNBC subtype. TNBC patients do not respond well to existing systemic therapies, since TNBC does not express any of the aforementioned biomarkers, and it exhibits an increased rate of recurrence. The lack of efficacious therapy and the propensity to develop metastasis are reflected in an overall survival for TNBC that has been stagnant over the past 20 years, with the median overall survival being less than 1 year after 1–2 lines of chemotherapy with rare meaningful responses [8,9].

Chemotherapy, the only treatment option for TNBC, uses drugs to destroy cancer cells. The most common first-line systemic TNBC therapy is a single-agent taxane, and the most common second-line regimens are capecitabine and combination gemcitabine–platinum therapy [10,11,12]. The Food and Drug Administration (FDA) in the US approved in 2018 the first targeted therapy, Olaparib, a poly(ADP-ribose) polymerase (PARP) inhibitor, for the treatment of metastatic HER2-negative breast cancer [13,14]. In 2019, the FDA approved Atezolizumab, the first immune checkpoint inhibitor for metastatic TNBC, expressing PD-L1 in combination with paclitaxel [15].

Metastatic breast cancer or TNBC is considered an incurable disease. Chemotherapy aims to control the cancer, prolong survival, and improve quality of life, but not to completely eradicate the disease. The general anti-tumor mechanism of chemotherapeutic agents is believed to involve binding to the tumor cell DNA, inhibiting cell division and DNA replication, leading to cancer cell destruction. The non-specificity of the drugs and susceptibility to multi-drug resistance cause adverse side effects in the human body during treatment, limiting its clinical applications.

In an attempt to overcome the adverse side effects and improve treatment outcomes, the combination of chemotherapy with other anti-cancer treatments, such as photodynamic therapy, has been demonstrated and explored further in the present study.

Photodynamic therapy (PDT) is a binary cancer treatment that involves delivery of a light-absorbing component, known as a photosensitizer (PS), to tumor tissues upon systemic administration, followed by visible light (600–800 nm) irradiation in the presence of endogenous oxygen [16,17]. Excitation of the PS in the red or near-infrared (NIR) region produces cytotoxic reactive oxygen species (ROS), such as singlet oxygen (^1^O_2_), to cause irreversible eradication of tumor cells and induce immune inflammatory responses and damage to tumor vasculature. PDT is an FDA-approved treatment for bronchial, esophageal, gastric, cervical, skin, head and neck cancers [18,19,20]. The effectiveness of PDT as both a therapeutic and palliative method for cancer is well documented [21].

In our laboratory, we synthesized a series of PDT agents that consist of light-activatable chlorin, which is derived from the plant-based molecule chlorophyll, chemically conjugated to vitamins or vitamin analogues [22,23,24]. In the present study, chemophotodynamic therapy (chemoPDT) or photochemotherapy, which is a combination of chemotherapy and photodynamic therapy, is presented. Chemotherapeutic agents, namely, taxol, doxorubicin, cisplatin, fluorouracil, and methotrexate, were each combined with our synthesized chlorin–vitamin conjugates as PDT agents, and their combined photodynamic efficacy was evaluated in a triple-negative breast cancer cell line. Drug combination is the most widely used technique to treat dreadful diseases such as cancer. The main purpose of the combination is to achieve a synergistic therapeutic effect, as well as a dose and toxicity reduction, thereby diminishing or delaying induction of drug resistance [25]. Synergism in binary therapy can cause minimization of toxicity effects and drug resistance.

## 2. Results and Discussion

### 2.1. Molecular Structures of Photosensitizers and Chemotherapeutic Drugs

**Figure 1:**
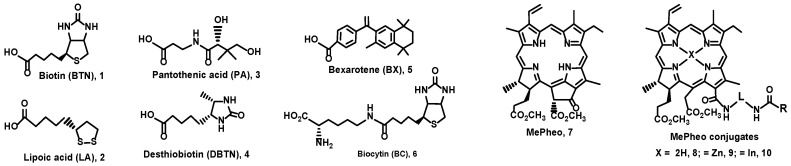
Molecular structures of vitamins and vitamin analogues. R: carbon chain of vitamin/analogue without CO_2_H from biotin CBTN, bexarotene CBX, lipoic acid CLA, pantothenic acid CPA, desthiobiotin CDBTN, and biocytin CBC; L: -(CH_2_)_6_-; X = H, Zn, In.

**Figure 2:**
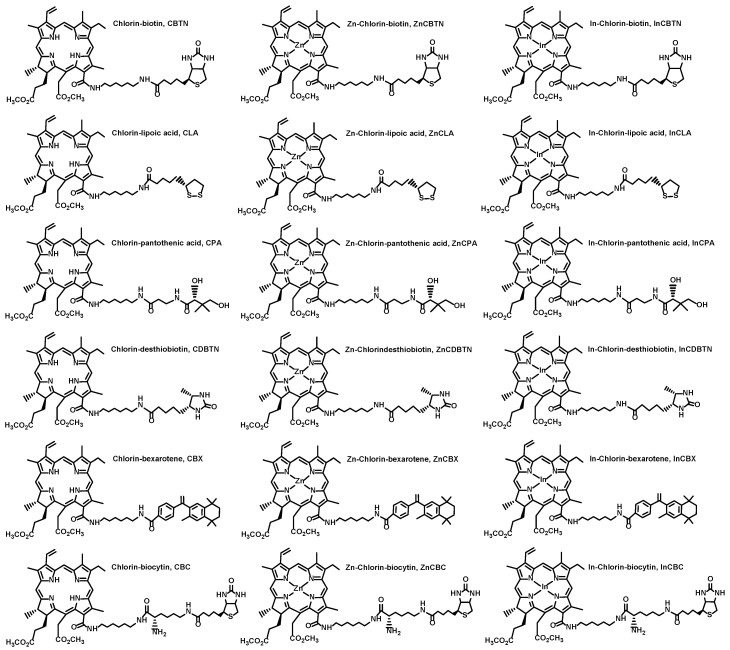
Molecular structures of synthesized photosensitizers linked to vitamins or vitamin analogues.

The molecular structures of the vitamins and analogues, namely, biotin (Vit H or B7) **1**, lipoic acid (coenzyme) **2**, pantothenic acid (Vit B5) **3**, desthiobiotin (biotin analogue) **4**, bexarotene (Vit A analogue) **5**, and biocytin (biotin metabolite) **6** are shown in Figure 1. The chlorin–vitamin conjugates were prepared by linking a chlorin (chlorophyll derivative) methyl pheophorbide a (MePheo) **7** and a vitamin (or vitamin analogue) to create MePheo-conjugates **8** and their Zn and In complexes **9**–**10** [22,23]. These series of compounds were covered by two patents and the chemical syntheses are published elsewhere [26,27]. The molecular structures of these compounds and the corresponding abbreviations are listed in Figure 2.

Co-treatment of the TNBC cells consisted of 24 h treatment with the chlorin–vitamin conjugates and each of the five known chemotherapeutic agents (paclitaxel, doxorubicin, cisplatin, fluorouracil, and methotrexate). The co-treated cells were then subjected to light treatment, and a cell survival assay was performed to determine the effect of the binary therapy.

**Figure 3:**
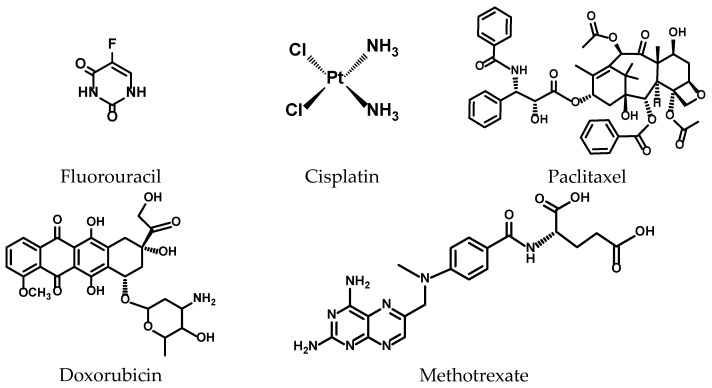
Molecular structures of chemotherapeutic agents.

The molecular structures of the chemotherapeutic agents are shown in Figure 3. Paclitaxel, doxorubicin, cisplatin, 5-fluorouracil, and methotrexate were purchased from Fisher Scientific. Safety Data Sheets (SDS) and toxicological information for the five chemotherapeutic compounds used are provided in the Supporting Information (Table S1).

### 2.2. Cell Viability after Combination Treatment in Triple-Negative Breast Cancer Cells

The synthetic photosensitizers used in this study were composed of methyl pheophorbide (MePheo), a chlorin derivative, conjugated to vitamins or vitamin analogues with their corresponding indium and zinc complexes, as listed in Figure 1. Our previous studies indicated a low photodynamic effect exhibited by the zinc complexes. Therefore, only the unmetallated PS and the indium complexes were included in this investigation. The light dose in this in vitro study corresponded to an energy fluence rate of 0.96 J/cm^2^ and a power of 16 mW/cm^2^.

**Figure 4:**
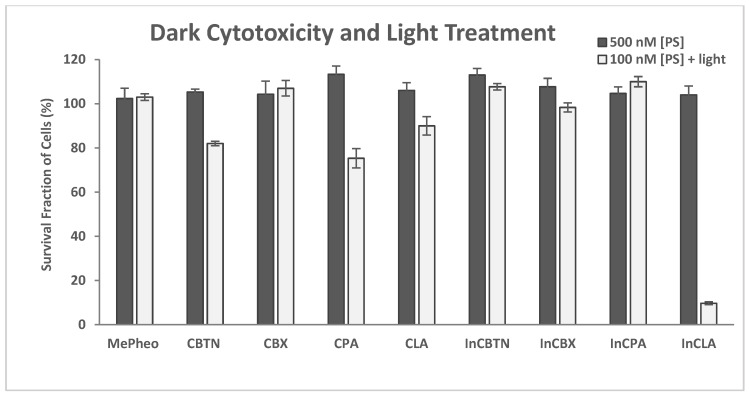
Cell survival assay of TNBC cells treated with photosensitizers at 500 nM in the dark and at 100 nM in the presence of light. Data reported are the mean ± SD of triplicate measurements in three separate experiments.

**Figure 5:**
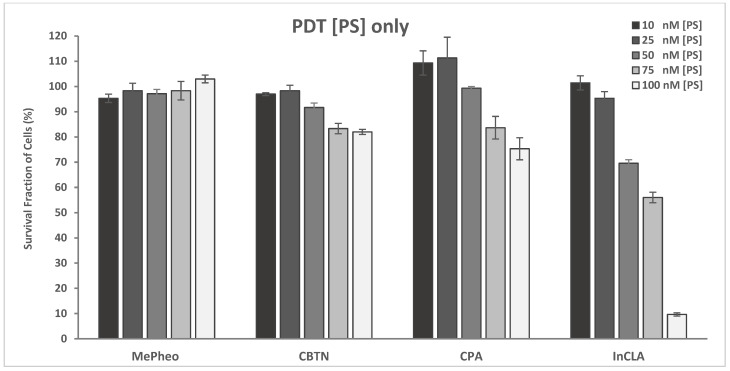
Cell survival assay of TNBC cells treated with photosensitizers (MePheo, CBTN, CPA, or InCLA) at varying concentrations of 10–100 nM followed by light exposure. Data reported are the mean ± SD of triplicate measurements in three separate experiments.

The dark cytotoxicity and light treatment of the PS-treated TNBC cells are shown in Figure 4. The PSs were CBTN, CBX, CPA, CLA, and their In complexes, including the starting precursor MePheo. The PS concentration was 500 nM and 100 nM in the dark and light-treated conditions, respectively. No cytotoxicity was observed in the absence of light, and InCLA (9.6% cell survival) was the most phototoxic, followed by CPA (75.3%) and CBTN (82.0%) at 100 nM. The TNBC cell survival at low concentrations (10–100 nM) of selected PSs (CBTN, CPA, InCLA, and MePheo) is shown in Figure 5, with InCLA exhibiting a significant dose-dependent response compared to the other PSs considered.

#### 2.2.1. PDT and Taxol

**Figure 6:**
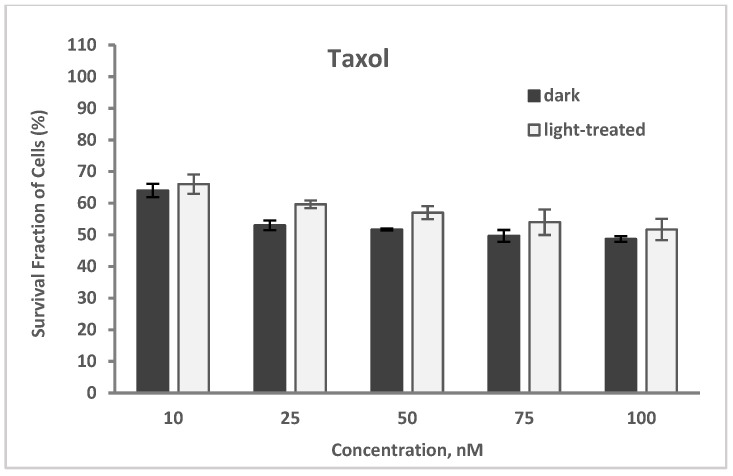
Cell survival assay of TNBC cells treated with taxol at varying concentrations of 10–100 nM in the dark or in the presence of light. Data reported are the mean ± SD of triplicate measurements in three separate experiments.

The cytotoxicity of taxol (paclitaxel) at varying concentrations of 10–100 nM in TNBC cells in the dark and upon light exposure is shown in Figure 6. The cell survival decreased from 64% to 49% and from 66% to 52% for the taxol-only treated cells in the dark and upon light irradiation, respectively. A very low increase (average of 4%) in cell survival was observed with the taxol-treated cells upon light irradiation compared to the unirradiated cells.

**Figure 7:**
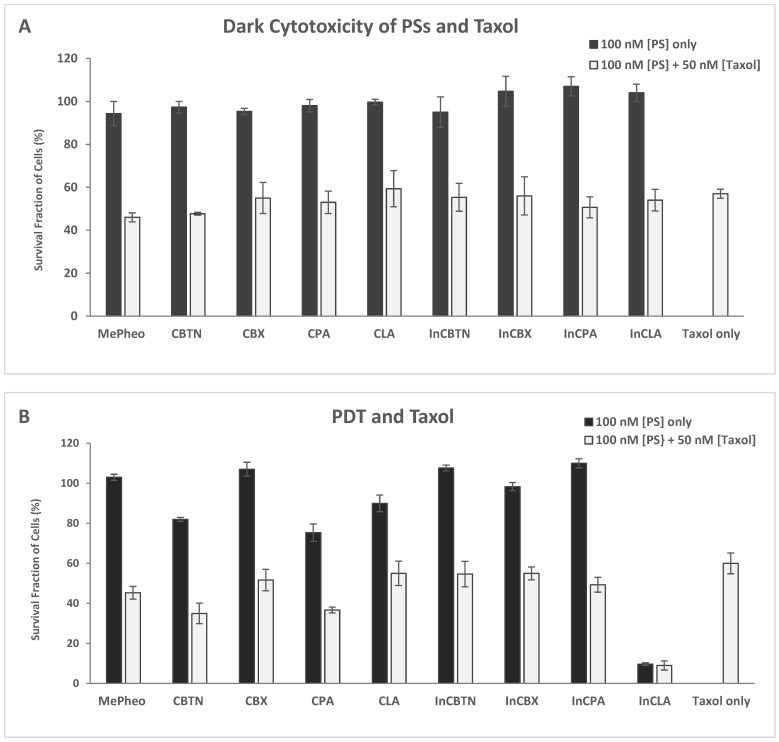
Cell survival assay of TNBC cells treated with photosensitizers at 100 nM and at 100 nM co-treated with 50 nM taxol in the dark (**A**), in the presence of light (**B**), and compared to taxol-only treated cells. Data reported are the mean ± SD of triplicate measurements in three separate experiments (n = 9) (*p* < 0.045, two-tailed *t*-test). InCLA at 100 nM showed an insignificant effect upon combination with taxol in the presence of light.

The PS-treated cells (at 100 nM) in the dark showed no cytotoxicity (Figure 7A). Co-treatment of the TNBC cells with PS (100 nM) and taxol (50 nM) for 24 h without light exposure resulted in an average of 53% cell survival (46% lowest for MePheo and 59% highest for CLA) compared to the taxol-only treated cells with 57% survival. In the absence of light, only the cytotoxic effect of taxol was effective.

**Figure 8:**
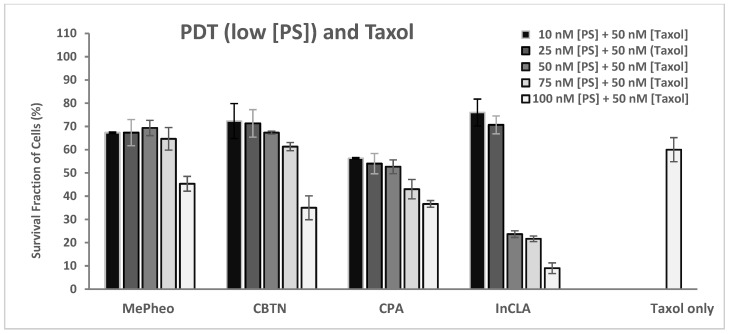
Cell survival assay of TNBC cells treated with photosensitizers (MePheo, CBTN, CPA, and InCLA) at varying concentrations of 10–100 nM and co-treated with 50 nM taxol in the presence of light and compared to taxol-only treated cells. Data reported are the mean ± SD of triplicate measurements in three separate experiments.

Upon irradiation with light of the cells co-treated with 50 nM taxol (Figure 7B), InCLA (at 100 nM) was the most phototoxic, with an observed 10% cell survival, followed by CBTN and CPA, with 35% and 37% cell survival, respectively. At the concentration of 100 nM, the photodynamic effect of InCLA prevailed and overpowered the effect of taxol, producing an insignificant effect from the combination. Except for InCLA, the average decrease in cell viability of the TNBC cells was 50% upon co-treatment with 50 nM taxol compared to PS-treatment alone at 100 nM. There was an average of a 7% decrease between combination therapy compared to taxol treatment alone (Figure 7B). The best PSs (MePheo, CBTN, CPA, and InCLA) that responded to co-treatment with taxol were tested at lower concentrations of 10–100 nM, as shown in Figure 7. At a lower concentration of 50 nM for both InCLA and taxol, the photodynamic cell destruction dramatically increased, as indicated by the amount of cells surviving from 70% for the InCLA-only treatment (Figure 5) to 24% in the presence of both InCLA and taxol (*p* = 0.003), with a decrease of 46% indicating a significant synergistic effect in TNBC cells (Figure 8). Compared to the taxol-only treated cells, binary treatment decreased the cell viability from 60% to 24%, which was a 36% decrease (*p* = 0.03). Thus, this observation suggests that combination therapy (InCLA and taxol) appears to be better than monotherapy alone.

Paclitaxel, which belongs to the taxane family and is sold under the brand name taxol, is a chemotherapy medication used to treat a variety of tumors (breast, lungs, pancreatic, ovarian, esophageal, cervical, Kaposi’s sarcoma). It is administered intravenously or as an albumin-bound formulation. The mechanism of therapeutic action of paclitaxel is based on its ability to bind to microtubules and promote the assembly of alpha and beta tubulin subunits, which are the building blocks of microtubules. The drug reduces the critical concentration of tubulin required for its assembly, promoting the lengthening of the tubulin polymer. The stability of the microtubules interferes with the dynamics of the microtubules, disrupting the ability of the cells to divide due to insufficient requirements of the mitotic checkpoint, halting cell division at the G2 or M phase [28]. Paclitaxel causes cytoskeletal abnormalities in which the microtubules become stubby and straight as opposed to fine sinuous filaments. The altered microtubules dislodge ribosomes off the rough endoplasmic reticulum (ER) and fuse nearby ER complexes together. Paclitaxel interferes with the dynamics of microtubule polymerization, delays the progression of mitosis by inducing the failure of chromosomal segregation, eventually leading to induction of apoptosis and mitotic arrest [29]. Not only via control of microtubule polymerization does paclitaxel affect human cells but also through Bcl-2 phosphorylation, mitochondrial calcium efflux and influx, and modulation of miRNA expression profiles [30]. A potential non-mitotic mechanism of paclitaxel action has been suggested, such that paclitaxel-induced rigid microtubules act to break the malleable cancer nuclei into multiple micronuclei. Tumor cells have a less sturdy and more pliable nuclear envelope than healthy cells due to the loss or reduction of lamin A/C proteins, providing paclitaxel selectivity to break the nuclear membrane and kill cancerous cells over non-neoplastic cells [31].

Previous studies involving in vitro and in vivo mouse models have been shown to synergize the effect of PDT with paclitaxel or platinum-based agents and reverse chemoresistance [32]. Tested in hepatocellular carcinoma, a light-triggered nanoplatform containing a red-emissive AIE-gen PS (labeled as TPA-BDTO) co-loaded with paclitaxel was prepared to create a nanoconstruct of TB/PTX@RTK for on-command drug release and synergistic chemophotodynamic therapy (chemo-PDT). The results of this study indicated that TB/PTX@RTK micelles (5 μg/mL in vitro) could specifically accumulate at the tumor site through the cRGD-mediated active target. Upon light irradiation, the AIE photosensitizer of TB could produce ROS, cleaving the thioketal linker to release PTX in tumor cells. This treatment also caused the upregulation of the expression of PD-L1 on the tumor cell surface. Thus, this study showed that chemo-PDT could synergistically inhibit tumor growth, and induce immunogenic cell death, eliciting an anti-tumor immune response.

Another study that used a prodrug composed of paclitaxel conjugated to a silicon phthalocyanine PS showed phototoxicity upon illumination with a 690 nm diode laser (5.6 mW/cm^2^ for 30 min) against SKOV-3 ovarian cancer cells at concentrations of 4–24 nM [33]. This synthesized prodrug was photochemically cleaved, releasing an alcohol derivative of paclitaxel in the presence of singlet oxygen that contributed to its cytotoxicity, with a 23-fold reduction in the IC_50_ value. This published study used a lower PS concentration of 4–24 nM and a power of 5.6 mW/cm^2^ for 30 min for light irradiation compared to the concentration of 50–100 nM used in our study using a power of 16 mW/cm^2^ for 1 min. The conditions used in our study are comparable to this published phototoxicity study.

In contrast, another study demonstrated that combining benzoporphyrin derivative (BPD) and paclitaxel is slightly antagonistic, with a combination index, CI, equal to 1.1 in glioblastoma U87 cells [34]. The combination of BPD and paclitaxel significantly downregulated Bcl-2 expression (*p* < 0.01), regulating apoptosis, but it did not significantly downregulate YAP, TAZ or EGFR expression in U87 cells (*p* > 0.05). Bcl-2 belongs to a group of regulatory proteins controlling apoptosis. Yes-associated protein (YAP)–transcriptional enhanced associate domain (TEAD) complex formation, TAZ (transcriptional co-activator with PDZ-binding motif) and EGFR (epidermal growth factor receptor) play important roles in proliferation, survival, and differentiation in both development and normal physiology, as well as in pathophysiological conditions.

Our results concerning the combination of the PS InCLA and paclitaxel produced a synergistic effect in terms of photodynamic efficacy more than the other PSs. Taxol is a microtubule-stabilizing agent that stimulates the polymerization of microtubules, suppressing their highly dynamic behavior and preventing dividing cells from progressing from metaphase into anaphase, eventually leading to apoptosis. The observed synergism between the two modalities could be explained by a combination of several mechanisms. (1) InCLA–PDT is in itself demonstrated to be cytotoxic to target TNBC cells. (2) InCLA-PDT can disrupt the cellular architecture, creating tumors that are more vulnerable to paclitaxel. A high cellular density is a critical barrier to the localization and penetration of chemotherapeutic agents in tumors. Treatment-induced apoptosis has specifically been shown to decrease the cellular density and enhance the uptake of chemotherapies into tumors. Therefore, InCLA–PDT-mediated apoptotic disruption of the membrane architecture could be important in improving the delivery of taxane agents into TNBC cells to achieve the best therapeutic benefit. (3) At the sub-cellular level, InCLA–PDT sensitizes TNBC cells to nuclear apoptotic signaling initiated by taxol, thereby lowering the threshold required to achieve a cytotoxic effect. The synergism exhibited by the combination could be driven by the ability of InCLA–PDT to disrupt the structure of the TNBC cell microstructural architecture, in addition to sensitizing the cells to apoptotic signals from taxol treatment. Evidence of apoptotic TNBC cells upon individual treatment of InCLA and taxol was observed, and during the combined treatment, indicating that increased apoptosis was the mechanism causing synergy. Further investigation into the alterations in protein expression following each monotherapy and combination treatment is warranted to fully understand the synergistic effect demonstrated by the binary therapy using PS–vitamin conjugates and paclitaxel.

#### 2.2.2. PDT and Doxorubicin

**Figure 9:**
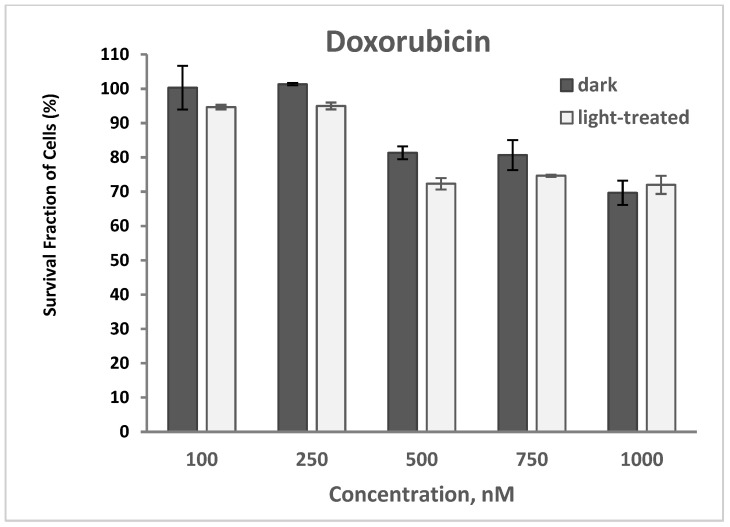
Cell survival assay of TNBC cells treated with doxorubicin at varying concentrations of 100–1000 nM in the dark or in the presence of light. Data reported are the mean ± SD of triplicate measurements in three separate experiments.

The cytotoxicity of doxorubicin (DOX) at varying concentrations of 100–1000 nM in TNBC cells in the dark and upon light exposure is shown in Figure 9. Cell survival decreased from 100% to 70% and from 95% to 72% for the doxorubicin-only treated cells in the dark and upon light irradiation, respectively. A very low decrease (average of 2–5%) in cell survival was observed with the doxorubicin-treated cells upon light irradiation compared to the unirradiated cells.

**Figure 10:**
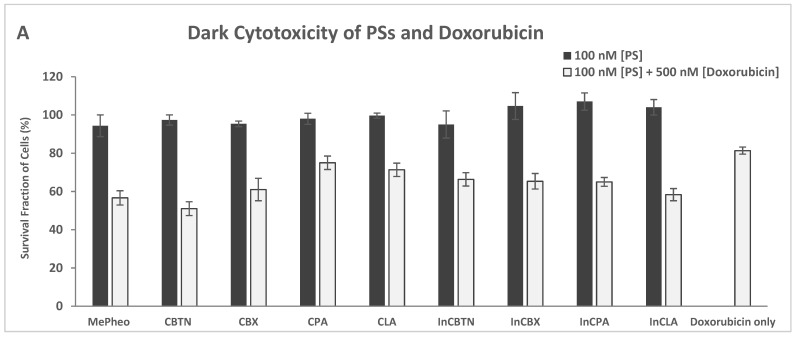

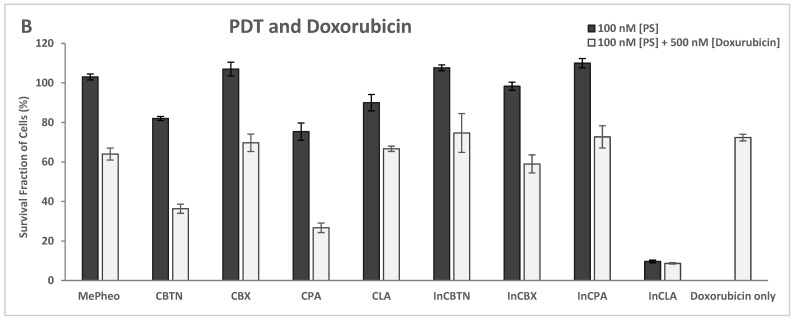
Cell survival assay of TNBC cells treated with photosensitizers at 100 nM and at 100 nM co-treated with 500 nM doxorubicin in the dark (**A**), in the presence of light (**B**), and compared to doxorubicin-only treated cells. Data reported are the mean ± SD of triplicate measurements in three separate experiments.

The PS-treated cells (at 100 nM) in the dark showed no cytotoxicity (Figure 10A). These are the same data as shown in Figure 7A, which are included here for comparison. Co-treatment of TNBC cells with PS (100 nM) and doxorubicin (500 nM) for 24 h without light exposure resulted in an average of 63% cell survival (51% lowest for CBTN and 75% highest for CPA) compared to the doxorubicin-only-treated cells with 81% survival. In the absence of light, only the cytotoxic effect of doxorubicin was involved in cell destruction.

**Figure 11:**
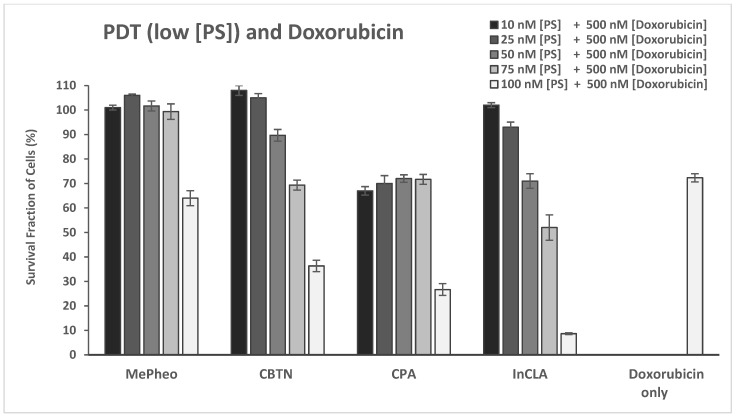
Cell survival assay of TNBC cells treated with photosensitizers (MePheo, CBTN, CPA, and InCLA) at varying concentrations of 10–100 nM and co-treated with 500 nM doxorubicin in the presence of light, and compared to doxorubicin-only treated cells. Data reported are the mean ± SD of triplicate measurements in three separate experiments.

Upon light exposure of the cells co-treated with 500 nM doxorubicin (Figure 10B), InCLA (at 100 nM) was still the most phototoxic, with an observed 9% cell survival, followed by CBTN and CPA, having 35% and 27% cell viability, respectively. InCLA still overwhelmed the effect of doxorubicin at this concentration. Lower concentrations (10–75 nM) of InCLA with 500 nM doxorubicin did not show an additive or synergistic effect upon co-treatment (Figure 11). CBTN and CPA at 100 nM showed a better photodynamic effect when co-treated with doxorubicin than PS-treatment alone. CBTN decreased from 82% to 36% when co-treated with DOX (*p* = 0.005), while CPA went from 75% to 27% (*p* = 0.02), with an average cell viability reduction of 47% for both PSs. When compared with DOX-treatment alone, the CBTN–DOX and CPA–DOX treatments decreased the cellular viability from 72% to 36% (*p* = 0.003) and 72% to 27% (*p* = 0.007), respectively. Thus, CBTN– or CPA–DOX PDT binary treatment was observed to be better than monotherapy alone, but at a higher concentration than that observed for InCLA-taxol PDT combination.

Doxorubicin (DOX), an anthracycline antibiotic, is the treatment of choice as a first-line chemotherapy for metastatic breast cancer patients not previously treated with anthracyclines. One of the mechanisms proposed for the anti-cancer activity of doxorubicin is the intercalation into the DNA as other anthracyclines, leading to the disruption of DNA repair. It inhibits topoisomerase II supercoiling the DNA during transcription, preventing DNA recombination of the double helix and blocking DNA replication [35]. Another mechanism proposed is the generation of free radicals damaging the DNA, which causes tumor cell destruction [36]. Another suggestion of the possible implications for mechanisms of cell killing during chemotherapy is that doxorubicin intercalation promotes nucleosome turnover around promoters due to its effect on DNA topology [37]. Doxorubicin causes DNA double-strand breaks in rapidly dividing cells, but whether it also causes an effect in the general chromatin properties is not known.

Previous studies involving in vitro and in vivo mouse models on the effect of combination of PSs with doxorubicin for chemophotodynamic therapy applications indicated either a synergistic, antagonistic or additive effect depending on the type and concentration of the photosensitizer and chemotherapeutic agent. In one particular study conducted at Roswell Park [38], (methyl-3-(1′-*meta*-iodo-benzyloxy) ethyl-3-devinylpyropheophorbide-a (PS1) in combination with doxorubicin as a single dose enhanced the long-term cure in SCID mice bearing small cell lung cancer (SCLC) 14,541 tumors. A Bliss model was generated to compare cell viability data from the monotherapy with those obtained by binary modality at various PS and DOX doses. The model generated a topological map by graphing 36 different peaks of antagonism, followed by valleys of synergy. A combination index of less than 1 indicated synergy, while for values greater than 1, the combination was antagonistic. The in vitro model suggested that for the best efficacy, DOX and PS should be used at a molar ratio of 2:1. In the binary therapy, the cell viability was reduced to 50% at 20 nM PS1 and 10 nM DOX, whereas PS1 alone at 20 nM yielded 90% viability and DOX alone at 10 nM showed 80% cell viability. While this synergistic effect was observed, no further explanation was offered as to the reason for such synergism caused by the combination of PDT and DOX.

Another study was conducted to investigate the effects of DOX and ZnPcS-PDT, alone and in combination, on BC cells [39]. Monotherapy with DOX and ZnPcS–PDT indicated inhibition of MCF-7 cells in a dose-dependent manner, with an optimal inhibitory concentration (IC_50_) of 0.9 and 1.1  µM, respectively. Cells were co-treated with a constant (IC_30_) dose of DOX (0.5  µM) and increasing concentration of ZnPcS-PDT (0.25, 0.5 and 1  µM). The results of this combination showed a significant reduction in the cell viability, with highest inhibition seen when 0.5  µM DOX and 1  µM ZnPcS–PDT were used. This study suggested that a lower dose of DOX when combined with PDT has a significant synergistic effect on MCF-7 cancer cells caused by the increased production of apoptotic cells. Again, no further mechanistic explanation was provided as to the enhanced effect of binary treatment.

Similarly, an investigation of the photosensitizing potential of DOX on murine L929 cancer cells showed an additional phototoxicity, with an absorption maximum of 579  nm. An increased cytotoxic mechanism of DOX was observed to be dependent on the increased production of oxygen radicals. Moreover, unique ^1^O_2_-dependent PDT cytotoxic effects have provided a further rationale for its combination with DOX to enhance the anti-tumor effect without overlapping toxicity [40].

Given the aforementioned studies on PDT combined with DOX, it is possible that in our study, the generation of more singlet oxygen induced by DOX might be the primary reason for the enhanced CBTN and CPA photodynamic activity. In the presence of light, DOX could trigger singlet oxygen formation due to its planar conjugated structure absorbing visible light, providing more energy to convert the ground state oxygen to the excited state, thus increasing the PS photodynamic efficacy against the TNBC cell line. Evidence of enhanced apoptosis was not observed in our study with the binary treatment with DOX, as illustrated later in this manuscript. Hence, further studies are needed to investigate if enhanced singlet oxidation production is indeed triggered by the combination treatment.

#### 2.2.3. PDT and Cisplatin

**Figure 12:**
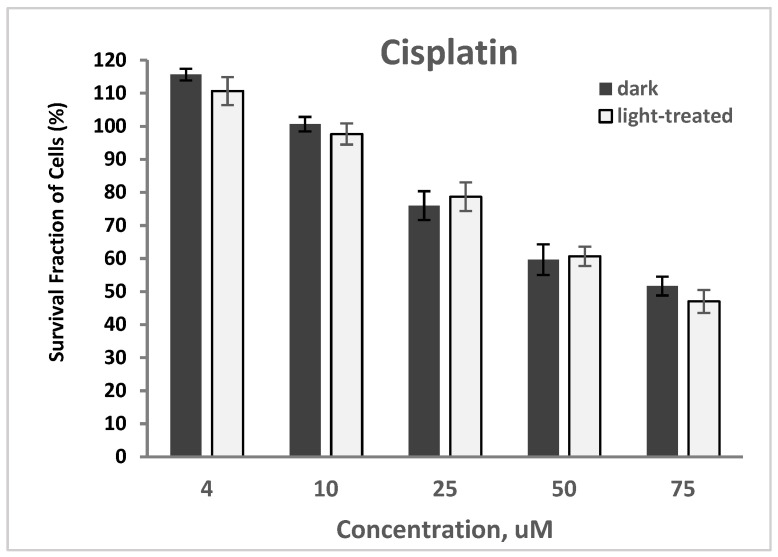
Cell survival assay of TNBC cells treated with cisplatin at varying concentrations of 4–75 μM in the dark or in the presence of light. Data reported are the mean ± SD of triplicate measurements in three separate experiments.

The cytotoxicity of cisplatin at varying concentrations of 4–75 nM in TNBC cells in the dark and upon light exposure is shown in Figure 12. Cell survival decreased from >100% to 52% and from >100% to 47% for the cisplatin-only treated cells in the dark and upon light irradiation, respectively. A very low decrease (average of 3%) in cell survival was observed with the cisplatin-treated cells upon light irradiation compared to the unirradiated cells.

The PS-treated cells (at 100 nM) in the dark showed no cytotoxicity (Figure S1A, Supplementary Materials), which represents the same data as shown in Figure 7A for comparison. Co-treatment of TNBC cells with PS (100 nM) and cisplatin (25 μM) for 24 h without light exposure did not result in any significant alteration in cellular viability compared to the cisplatin-only-treated cells with 72% survival (Figure S1A). In the absence of light, the presence of cisplatin in the TNBC cells caused no effect.

**Figure 13:**
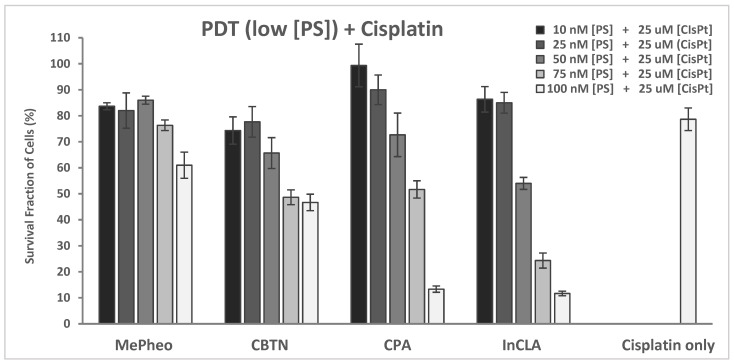
Cell survival assay of TNBC cells treated with photosensitizers (MePheo, CBTN, CPA, and InCLA) at varying concentrations of 10–100 nM and co-treated with 25 μM cisplatin in the presence of light, and compared to cisplatin-only treated cells. Data reported are the mean ± SD of triplicate measurements in three separate experiments.

Upon light exposure of the cells co-treated with 25 μM cisplatin (Figure S1B, Supplementary Materials), InCLA (at 100 nM) remained the most phototoxic, with an observed 10% cell survival, followed by CPA and CLA, having 13% and 35% cell viability, respectively. InCLA also overwhelmed the effect of cisplatin at this concentration. CPA and CLA at 100 nM showed a better photodynamic effect when co-treated with cisplatin than PS-treatment alone. CPA decreased from 75% to 13%, while CLA went from 90% to 35%, with an average decline of 59% in cell survival for both PSs. Except for InCLA, the decrease in cell viability of the TNBC cells ranged from 62% to 3% upon co-treatment with 25 μM cisplatin compared to PS-treatment alone at 100 nM. There was an average of a 28% decrease between the binary treatment with PS and cisplatin compared to PS or cisplatin treatment alone (Figure S1B). However, when compared to the cisplatin-treated cell viability, the effect of added cisplatin only caused reduced cell survival in the MePheo-, CBTN-, CPA-, and CLA-co-treated TNBC cells. At 100 nM, CPA-cisplatin treated cells produced a significant decrease in cell viability when compared either with CPA–PDT monotherapy from 79% to 13% (*p* = 0.008) or cisplatin monotherapy from 75% to 13% (*p* = 0.005). At lower concentrations of selected PSs (MePheo, CBTN, CPA and InCLA) at 50 nM co-treated with 25 μM cisplatin, a reduced cell survival ranging from 28% to 11% was observed, with an average of 23%, as shown in Figure 13, compared to the PS-treated only cells in Figure 7B. At a lower concentration of 50 nM for InCLA and 25 μM cisplatin, the photodynamic cell destruction increased, as indicated by the amount of cells surviving from 70% for InCLA-only treatment (Figure 5) to 54% in the presence of both InCLA and cisplatin (*p* = 0.04), with a decrease of 16% indicating a nearly additive effect in TNBC cells (Figure 8). Compared to the cisplatin-only treated cells, the binary treatment of InCLA–PDT–cisplatin decreased cell viability from 79% to 54%, which was a 25% decrease (*p* = 0.05). Thus, this observation suggests that the InCLA–cisplatin or CPA–cisplatin combination appears to be better than InCLA–PDT or CPA–PDT or cisplatin monotherapy alone.

Cisplatin or cisplatinum, also called *cis*-diamminedichloroplatinum(II), is a metallic Pt coordination compound with a square planar geometry. It is approved for clinical treatment of various cancers. Its molecular mechanism of action primarily involves formation of DNA adducts, leading to the onset of programmed cell death through initiation of the major signaling pathways. Production of free radicals from the ruptured cell membrane due to the disruption of calcium homeostasis, lipid peroxidation, DNA damage, p53 activation, overexpression of p38, MAPK and JNK signaling pathways, caspase 3 and caspase 9 activation, and downregulation of oncogenes collectively triggers apoptosis of cancer cells [41,42]. Once inside the cell, the activated form of cisplatin is a product of the reaction of water in the cytoplasmic compartment of the cell. The hydrolyzed product is a potent electrophile that can react with nucleophiles such as sulfhydryl groups on proteins and nitrogen donor atoms on nucleic acids [43]. Cisplatin binds to the N7 reactive center of purine residues and can cause DNA damage in cancer cells, blocking cell division and resulting in apoptotic cell death. The 1,2-intrastrand cross-links of purine bases with cisplatin are the most notable among the changes in DNA. These include the 1,2-intrastrand d(GpG) and 1,2-intrastrand d(ApG) adducts representing about 90% and 10% of adducts, respectively, contributing to cisplatin cytotoxicity. These molecular mechanisms of cytotoxicity constitute the hallmarks of cisplatin bioactivity [44]. BRCA-mutated, sporadic triple-negative or basal-like breast cancer displays aberrant DNA repair and genomic instability, stipulating the rationale for using platinum-based entities that provoke DNA damage.

Studies conducted in vitro on NCI-H446 small-cell lung cancer (SCLC) cells co-treated with low-dose cisplatin (1 μM) and PDT (1.25 J/cm^2^) synergistically inhibited cell viability and cell migration [45]. This same study showed that the combined therapy induced a higher level of intracellular ROS in cultured NCI-H446 cells. Additionally, the synergistic effect by cisplatin and PDT was recapitulated in the tumor xenograft in a nude mouse model, as revealed by an increase in the induction of apoptosis/necrosis and decrease in tumor volume. Cisplatin triggers endoplasmic reticulum (ER) stress, mitochondrial dysfunction, and subsequent production of reactive oxygen species.

Cell treatment with combined cisplatin–laser in another investigation showed that the combination of cisplatin and laser radiation can significantly reduce the cell viability percentage of both A2780 (cisplatin-sensitive) and A2780-CP (cisplatin-resistant) cervical cancer cell lines [46], and in a breast tumor-bearing nude mouse model treated with Photolon (as a photosensitizer) and cisplatin [47]. These results further support the effectiveness of combination therapy with various mechanisms leading to cancer cell destruction. Similarly, the combination effect of benzoporphyrin derivative (BPD) and cisplatin in glioblastoma U87 cells is slightly synergistic (combination index, CI = 0.9), showing 1.8- to 2.6-fold lower half maximal inhibitory concentrations (IC_50_) compared to those of the individual drugs alone. Another study [48] using BPD followed by carboplatin treatment in a 3D model of metastatic ovarian cancer observed a significant synergistic reduction compared to PDT or carboplatin alone. Reversing the order of treatment (carboplatin followed by BPD–PDT) did not produce synergism. In that particular study, the BPD and carboplatin concentrations utilized were 0.250 μM and 40 mg/m^2^, respectively, with a PDT dose of 1.25 μM·J/cm^2^ (0.250 μM BPD and 5.0J/cm^2^ of 690 nm light). The carboplatin concentration used for this reported in vitro study was one-tenth of the clinically effective dose for intraperitoneally administered carboplatin.

It is plausible that in our study with binary treatment using PDT and cisplatin, our synthesized PSs induced the production of ROS, which can be additionally enhanced by the presence of cisplatin, prompting the additive inhibitory effect on TNBC cells. Evidence of few apoptotic cells upon light irradiation after combination treatment might be the other reason for the enhanced photodynamic efficacy. Hence, additional studies are necessary to fully understand the mechanism of the increased effect upon co-treatment with PS and cisplatin.

#### 2.2.4. PDT and Fluorouracil

**Figure 14:**
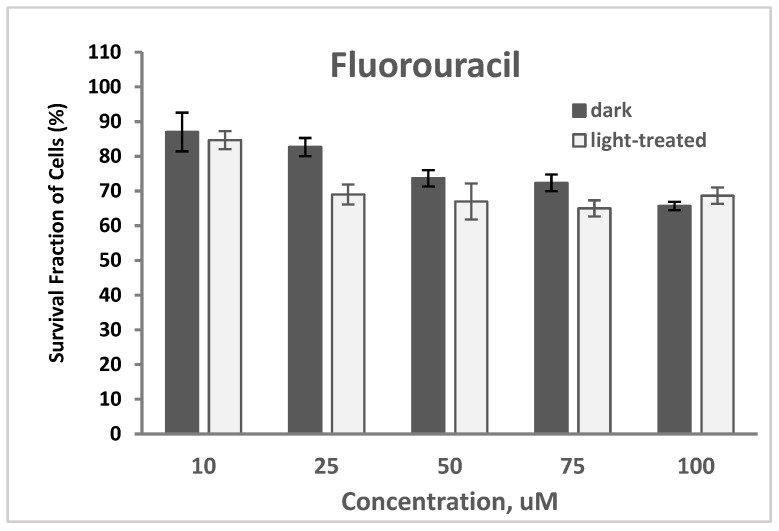
Cell survival assay of TNBC cells treated with fluorouracil at varying concentrations of 10–100 μM in the dark or in the presence of light. Data reported are the mean ± SD of triplicate measurements in three separate experiments.

The cytotoxicity of fluorouracil at varying concentrations of 10–100 nM in TNBC cells in the dark and upon light exposure is shown in Figure 14. Cell survival decreased from 87% to 65% and from 85% to 68% for the fluorouracil-only treated cells in the dark and upon light irradiation, respectively. A decrease in cell survival with an average of 9% was observed with the fluorouracil-treated cells upon light irradiation compared to the unirradiated cells.

The PS-treated cells (at 100 nM) in the dark showed no cytotoxicity (Figure S2A, Supplementary Materials), which represents the same data as shown in Figure 7A for comparison. Co-treatment of TNBC cells with PS (100 nM) and fluorouracil (25 μM) for 24 h without light exposure resulted in an average of 73% cell survival (61% lowest for MePheo and 84% highest for CLA) compared to the fluorouracil-only-treated cells with 64% survival. In the absence of light, only the cytotoxic effect of fluorouracil was involved in cell destruction (Figure S2A, Supplementary Materials).

**Figure 15:**
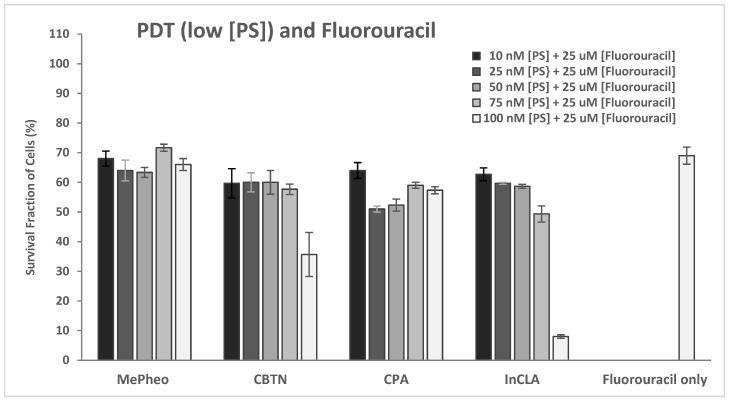
Cell survival assay of TNBC cells treated with photosensitizers (MePheo, CBTN, CPA, and InCLA) at varying concentrations of 10–100 nM and co-treated with 25 μM fluorouracil in the presence of light, and compared to fluorouracil-only treated cells. Data reported are the mean ± SD of triplicate measurements in three separate experiments.

Upon light exposure of the cells co-treated with 25 μM fluorouracil (Figure S2B, Supplementary Materials), InCLA (at 100 nM) still remained the most phototoxic, with an observed 8% cell survival, followed by CBTN and CPA, having 36% and 57% cell viability, respectively. InCLA also overwhelmed the effect of fluorouracil at this concentration. CBTN and CPA at 100 nM showed a better photodynamic effect when co-treated with fluorouracil than PS-treatment alone. CBTN decreased from 81% to 36%, while CPA went from 75% to 57%, with an average decline of 31% in cell survival for both PSs. Except for InCLA, the decrease in cell viability of TNBC cells ranged from 46% to 7% upon co-treatment with 25 μM fluorouracil compared to PS-treatment alone at 100 nM. There was an average of a 22% decrease between the combination therapy and PS treatment alone (Figure 7B). However, when compared to the fluorouracil-treated cell viability, the effect of added fluorouracil caused reduced cell survival in only the CBTN and CPA co-treated TNBC cells. At lower concentrations of selected PSs, only CBTN at 100 nM co-treated with 25 μM fluorouracil showed a significant cell viability reduction from 82% to 36% (*p* = 0.03), with a difference of 46%, as shown in Figure S2B, Supplementary Materials (or Figure 15), compared to the PS-treated only cells in Figure 7B. However, the effect was less significant when compared with the 5FU-treated cells (*p* = 0.06), even though a 33% reduction in cell viability was observed. CBTN as a PS in combination with fluorouracil was observed to be the best compared to the monotherapy alone and with the other PSs used.

Moreover, 5-fluorouracil (5FU) is the first rationally designed antimetabolite chemotherapeutic drug widely used, either alone or in combination with other drugs, to treat solid tumors of the digestive origins (gastric, esophageal, colorectal, anal, and pancreatic) and those arising in other organs (breast, cervix, and head and neck) [49]. The synthetic design of 5FU, a fluorinated pyrimidine, was based on the observation that tumor tissues utilize uracil more rapidly than normal tissues [50]. The anti-folate or anti-vitamin folic acid property of fluoropyrimidines is thought to be the principal mechanism of action. Fluoropyrimidines are intracellularly converted into the anti-folate 5-fluorodeoxyuridine monophosphate (5-FdUMP), forming a covalent intermediate with the folate-dependent enzyme thymidylate synthase (TS) [51]. An imbalance in the nucleotide pool affecting DNA synthesis, possibly through incorporation of uracil, results due to the inhibition of the conversion of 2′-deoxythymidine 5′-monophosphate (dTMP) from 2′-deoxyuridine 5′-monophosphate (dUMP). The impairment of nucleotide metabolism causes damage during genome replication, with negative consequences in rapidly dividing cells such as cancer cells. The key enzyme inhibited by 5FU is thymidylate synthase (TS), which is responsible for the de novo synthesis of dTMP [52]. Its uniqueness in targeting TS expression independent of the breast cancer status, and its cost-effectiveness compared to other chemotherapeutic drugs, make 5FU an attractive choice for BC treatment, even though 5FU is not without severe side effects and the emergence of acquired or inherent chemoresistance. A recent study demonstrated that 5FU treatment of a colorectal cell line resulted in the production of fluorinated ribosomes, referred to as F-ribosomes, causing extrinsic chemical ribosomal RNA (rRNA) modifications, altering its intrinsic translational activity that potentially could contribute to drug resistance, cellular plasticity and relapse post-5FU chemotherapy [53].

Furthermore, 5-fluorouracil is one of the most common chemotherapeutic drugs for TNBC. However, the toxicity of 5FU diminishes its therapeutic index. Thus, combination therapy using fluorouracil with natural products with potent anti-cancer activities, such as curcumin and β-elemene (ELE), has been investigated. A sub-optimal dose treatment of 5FU plus curcumin in TNBC xenograft models indicated a reduction in tumor-related parameters and showed the ability of phytochemical curcumin in chemosensitizing BC to 5FU [52]. β-elemene, which belongs to a group of natural compounds derived from medicinal plants and herbs such as Rhizomazedoariae and Curcuma, has been approved by the Chinese Drug and Food Administration to treat leukemia and liver, breast and brain carcinoma. An experimental study revealed that ELE enhanced the effect of 5FU against cell viability, proliferation, migration, invasion, and colony formation in the MDA-MB-231 and BT549 TNBC cell lines, and this combination treatment inhibited the tumor growth and modulated its molecular markers in mouse xenograft models [54].

Previous studies conducted in cultured MCF-10F (control) and MDA-MB-231 experimental BC cell models exposed to low doses of high linear energy transfer (LET) α-particle radiation of 150 keV/μm and treated with fluorouracil indicated that fluorouracil exerted apoptotic activity in breast cancer cells by regulating genes of the Ras family and related apoptosis, such as Bax, Bcl-xL and NF-κB expressions. Flow cytometry revealed 80% cell killing compared to 21.5% in the control MCF-10F cell line [55].

A clinical study that explored the mechanisms of action of neoadjuvantal 5FU and PDT for actinic keratosis verified that the protoporphyrin (PpIX, as a photosensitizer) levels increased 2- to 3-fold in 5FU-pretreated lesions versus controls. Altered expression of heme enzymes (coproporphyrinogen oxidase and ferrochelatase) and induction of p53 were observed and probably accounted for the enhanced PpIX levels, leading to cancer cell destruction [56]. Actinic keratoses are precancerous lesions that increase the skin cancer risk in patients with chronic sun damage and immunosuppression.

Another study showed that photosensitizers such as benzoporphyrin derivative (BPD), protoporphyrin IX and hematoporphyrin can inhibit Yes-associated protein (YAP)-transcriptional enhanced associated domain (TEAD) complex formation, which is responsible for promoting tumor progression, cancer proliferation, chemoresistance, and metastasis [57]. A later study also demonstrated that BPD inhibits YAP and sensitizes hepatocellular carcinoma cells in vitro to chemotherapeutic treatment with doxorubicin and fluorouracil through the autophagy-related cell death pathway [58].

It is possible that the effect observed in our binary treatment (100 nM CBTN co-treated with 25 μM fluorouracil) showing a cell viability reduction of 46% is the effect caused by the inhibition of YAP sensitizing the TNBC cells to fluorouracil treatment via an autophagy cell death mechanism. BPD has a similar macrocyclic core structure as the chlorin macrocycle in our synthesized CBTN. The fact that only CBTN produced a significant reduction in cell viability and not the other PS-vitamin conjugates indicated there is some specificity associated with the biotin linkage that enhanced the effect when combined with fluorouracil (an anti-folate drug) upon treatment. Both biotin and folic acid are key coenzymes involved in the fatty acid and nucleotide biosynthetic pathways, respectively. Further studies need to be pursued to validate the effect of PS and fluorouracil upon light irradiation and to investigate the expression levels of various proteins affected in the PDT-induced cell death mechanism in TNBC.

#### 2.2.5. PDT and Methotrexate

**Figure 16:**
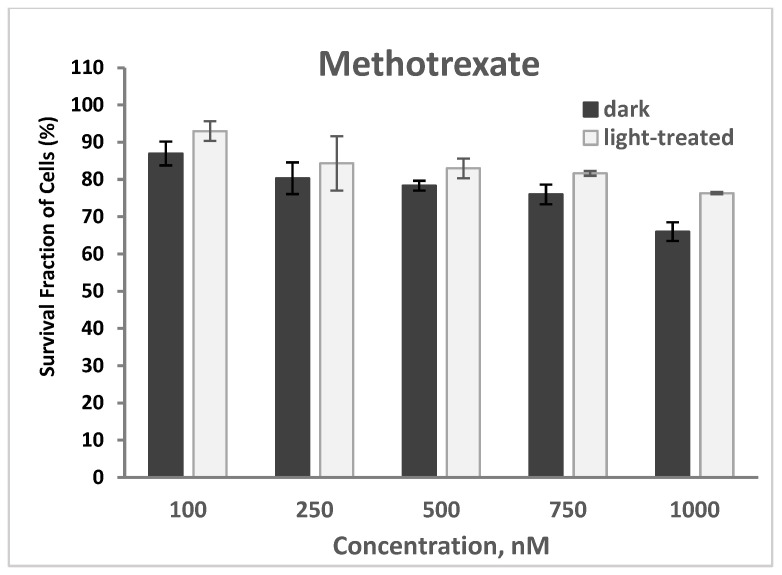
Cell survival assay of TNBC cells treated with methotrexate at varying concentrations of 100–1000 nM in the dark or in the presence of light. Data reported are the mean ± SD of triplicate measurements in three separate experiments.

The cytotoxicity of methotrexate at varying concentrations of 100–1000 nM in TNBC cells in the dark and upon light exposure is shown in Figure 16. Cell survival decreased from 87% to 66% and from 93% to 66% for the methotrexate-only treated cells in the dark and upon light irradiation, respectively. An increase (average of 6%) in cell survival was observed with the methotrexate-treated cells upon light irradiation compared to the unirradiated cells.

The PS-treated cells (at 100 nM) in the dark showed no cytotoxicity (Figure S3A, Supplementary Materials), which represents the same data as shown in Figure 7A for comparison. Co-treatment of TNBC cells with PS (100 nM) and methotrexate (MTX, 500 nM) for 24 h without light exposure resulted in an average of 72% cell survival (61% lowest for InCPA and 85% highest for CBTN) compared to the methotrexate-only-treated cells with 78% survival. In the absence of light, only the cytotoxic effect of methotrexate was involved in cell destruction (Figure S3A, Supplementary Materials).

**Figure 17:**
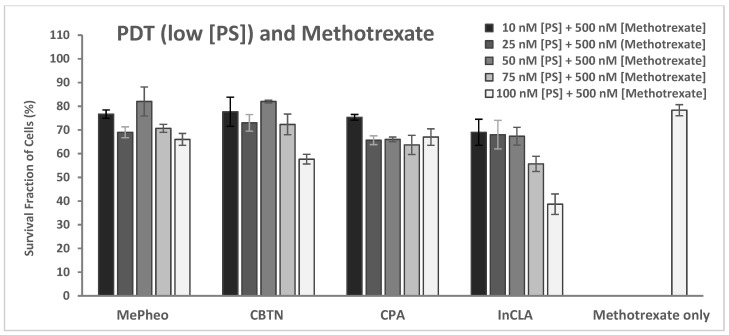
Cell survival assay of TNBC cells treated with photosensitizers (MePheo, CBTN, CPA, and InCLA) at varying concentrations of 10–100 nM and co-treated with 500 nM methotrexate in the presence of light, and compared to methotrexate-only treated cells. Data reported are the mean ± SD of triplicate measurements in three separate experiments.

Upon light irradiation of the cells co-treated with 500 nM methotrexate (Figure S3B, Supplementary Materials), InCLA (at 100 nM) still remained the most phototoxic among the PSs tested, with an observed 39% cell survival, followed by CLA or CBTN, CBX, and InCBX, having the same 57–58% cell viability. However, InCLA was observed to cause enhanced cell viability at this concentration. At lower concentrations of selected PSs, only CPA at 50 nM co-treated with methotrexate (500 nM) showed a significant cell viability reduction from 99% (Figure 5) to 66% (Figure 17), with a difference of 33% (*p* = 0.002), but it was not as significant when compared with the methotrexate-treated cells in which the CPA–PDT–MTX co-treatment only caused a 12% reduction in cell survival (*p* = 0.06). MePheo and CBTN were observed to cause a difference of only 10% in cell survival compared to PS treatment alone (Figure 17). CPA as a PS in combination with methotrexate appeared to be the best compared to monotherapy alone and with the other PSs used.

Methotrexate (amethopterin) is a synthetic structural analogue of folic acid created as a less toxic derivative of aminopterin, which is a folic acid antagonist used to treat children with acute leukemia. Methotrexate, also considered an anti-metabolite (anti-vitamin) of folic acid, indirectly inhibits cell division through the blockage of folate-related enzymes, mainly dihydroxyfolate reductase (DHFR), which catalyzes the conversion of dihydrofolate to tetrahydrofolate (THF). THF serves as a coenzyme in transmethylation reactions in the nucleotide biosynthetic pathways, which is essential in synthesis, repair or replication of DNA strands. Methyl-THF acts as a methyl donor in methylation reactions of DNAs, RNAs, proteins, phospholipids and amino acids syntheses. Methotrexate inhibition of intracellular THF production results in the disruption of cell proliferation, causing a metabolic imbalance [59]. Moreover, it also induces inhibitory effects on other enzymes, including thymidylate synthase (TYMS) [60], 5-aminoimidazole-4-carboxamide ribonucleotide transformylase (AICART) [61] and amido-phosphoribosyltransferase [62], participating in the de novo biosynthesis of purine and pyrimidine nucleotides. Methotrexate bioactivity is most visible in highly proliferating and actively dividing cancer cells, specifically in the *S* phase of the cell cycle, further indicating that the antagonism of folate is related to the anti-tumor activity of the drug. Cellular absorption of methotrexate uses folate receptors via the receptor-mediated endocytosis responsible for internalizing bound folates or folate conjugates [63]. Currently, methotrexate is commonly utilized for the treatment of various neoplastic diseases (breast, bladder, leukemia, osteosarcoma, and number of other cancers) and severe and resistant forms of autoimmune diseases (rheumatoid arthritis, psoriasis, Crohn’s disease, and multiple sclerosis, among others) [64].

The efficacy of combination therapy composed of doxorubicin (DOX) or methotrexate (MTX) with Photosense (AlPcS_2-4_), a PS containing a mixture of aluminum phthalocyanines with different degrees of sulfonation, was investigated in cervical Hela, breast MCF-7, and rat brain RG2 cell lines. The results revealed higher synergistic effects when DOX- or MTX-mediated chemotherapy preceded PDT light activation for 24 h. MTX and DOX exposure prior to AlPcS_2-4_ treatment may enhance mitochondrial PS localization, causing simultaneous targeting of DNA, proteins, and lipids that leads to high cytotoxicity at submicromolecular drug doses [65].

In a similar study, PDT with ALA (δ-aminolevulinic acid) was synergistically enhanced by combined therapy using MTX-preconditioned monolayer cultures of immortalized keratinocytes, mouse skin tumors and implanted human A431 squamous cell tumors in mice. MTX boosted the PpIX levels over a broad concentration range of 0.6 nM to 2 mM, correlating with changes in the protein expression of key porphyrin enzymatic pathways (coproporphyrinogen oxidase and ferrochelatase). Additionally, MTX selectively induced differentiation markers (E-cadherin, involucrin, and filaggrin) [66].

According to the results from our study of the combined treatment of the synthesized PSs and methotrexate, no PS demonstrated a dramatic reduction in cell viability. It was also observed that InCLA (100 nM) with methotrexate (500 nM) showed higher cell survival of 39% compared to InCLA alone (9.7%) in the presence of light. Methotrexate seemed to cause the TNBC cells to resist the effect of InCLA–PDT treatment. It is conceivable that this could be an effect of drug resistance and several factors may cause the TNBC cell line used to resist the effect of methotrexate. Since methotrexate is a potent inhibitor of dihydrofolate reductase (DHFR), a key enzyme required for intracellular folate metabolism and functions to regenerate tetrahydrofolate from dihydrofolate, a product of thymidylate synthase, these TNBC cells in our in vitro study could have a mutated DHFR, causing the methotrexate to bind more loosely than in the normal enzyme and thus lessening its chemotherapeutic effect [67]. In addition to the mutational effect, other factors that could come into play to explain the minimal effect of methotrexate include hypoxia, autophagy and overexpression of resistance genes or mutations in tumor suppression genes (i.e., *p53* gene). In our study, only CPA–PDT combined with MTX showed some significant photodynamic effect. Additional studies are necessary to further elucidate the mechanisms for the results observed in our investigation.

#### 2.2.6. Cell Viability Summary and Combination Index

Cell inhibition in TNBC cells is determined based on the cell survival (MTT) assay of the binary therapy blending the effect of PDT and chemotherapeutic agents. In this study, it should be noted that monotherapy alone, either PDT with the photosensitizers (with light) or chemotherapy with chemotherapeutic agents (with or without light), resulted in less treatment efficacy than combination treatment. Table 1 tabulates the percentage inhibition of TNBC cells treated with our eight synthesized PSs (CBTN, CBX, CPA, CLA, and their corresponding indium complexes) and the starting compound MePheo at 100 nM, and simultaneously treated with taxol (50 nM), doxorubicin (500 nM), cisplatin (25 mM), fluorouracil (25 mM), or methotrexate (500 nM). MePheo served as a control compound. The TNBC cells were then exposed to light after binary treatment for 24 h.

The order of inhibition for the nine PSs tested in this study from the most potent to the least potent chemophotodynamic efficacy is provided as follows:

For the PSs and taxol combination:

InCLA >> CBTN > CPA > MePheo > InCPA > CBX > CLA, InCBTN, InCBX

For the PSs and doxorubicin combination:

InCLA >> CPA > CBTN > InCBX > MePheo > CLA > CBX > InCPA > InCBTN

For the PSs and cisplatin combination:

InCLA > CPA > CLA > CBTN > MePheo > InCBX > CBX, InCBTN, InCPA

For the PSs and methotrexate combination:

InCLA >> CLA > CBTN, CBX, InCBX > MePheo > CPA, InCBTN > InCPA

For the PSs and fluorouracil combination:

InCBTN, InCPA > InCBX > CBTN > CPA > CLA > MePheo > InCLA > CBX

Based on these data of the PSs at a 100 nM concentration, InCLA exhibited the most potency, either alone or in combination, specifically with taxol, doxorubicin, cisplatin, and fluorouracil. Following InCLA were CPA and CBTN, having the best chemophotodynamic efficacy among the synthesized PSs tested. InCLA, CPA and CBTN showed better activity against TNBC cells than the starting compound MePheo. The best combination for CBTN was with taxol, followed by doxorubicin or fluorouracil, while for CPA, cisplatin was the best, next doxorubicin, then taxol.

The combination index (CI) for the binary treatment of TNBC cells with a PS and a chemotherapeutic drug was calculated based on the Chou–Talalay method, which is a mathematical system analysis of the dose–effect dynamics derived from the mass-action law (MAL) principle, a unified theory correlating single entity and multiple entities [68,69]. The Chou–Talalay algorithm utilized in this study is a freeware available online as CompuSyn software [70]. The interaction dynamics of multiple entities resulted in the combination index equation, and this computer simulation offered an automatic quantitative determination of the Synergism (CI < 1), Additive (CI = 1), and Antagonism (CI > 1). The unified dynamics algorithm used a minimum of dose data points to fit the general MAL theory.

The CI values for the PSs selected based on the best biological activity to inhibit the growth of TNBC cells in combination with the known chemotherapeutic entities used in this study are listed in Table 2, as calculated using CompuSyn software Version 1.0 (Last accessed 22 April 23). Our synthesized PSs proved to be better than the starting compound MePheo and confirmed our results in the cell viability assay. The combination of InCLA and taxol showed the strongest synergism with the lowest CI value of 0.25. Other combinations in the table range from synergistic to nearly additive, with CI values ranging from 0.41–1.09. The calculated CI values are based on a 50 nM concentration for the PSs, 50 nM taxol, 500 nM doxorubicin, 25 μM cisplatin, 25 μM fluorouracil, and 500 nM methotrexate.

Ranking the combinations based on the CI values in decreasing order:

InCLA: taxol > fluorouracil > cisplatin

CPA: taxol > fluorouracil > methotrexate

CBTN: fluorouracil > taxol > cisplatin

Based on the low nanomolar dosage used for the binary therapy, the best chemophotodynamic combination treatment against TNBC in decreasing order was the following:

InCLA + taxol > CPA + taxol > CBTN + taxol

The differences in the CI values and concentration differences at which an optimal effect was observed could also be attributed to the differential cell death mechanisms elicited by the chemotherapies (i.e., DNA-damaging cisplatin and microtubule-stabilizing paclitaxel), which could interact differently with the light-independent intracellular effects of the synthesized PSs.

### 2.3. Microscopy Studies

#### 2.3.1. Fluorescence Microscopy

PDT can initiate several forms of cell death pathways that include apoptosis, autophagy and/or necrosis. Apoptosis, or programmed cell death, is the most widely studied of the forms of cell death. Its morphological characteristics can be identified under light microscopy and include cell shrinkage, chromatin condensation, nuclear fragmentation, blebbing of the cytoplasmic membrane, loss of adhesion and cellular volume, and finally, the formation of apoptotic bodies [71,72]. Autophagy (or self-eating) is a conserved cellular degradation process that eliminates molecules and subcellular elements, including nucleic acids, proteins, lipids and organelles, via lysosome-mediated degradation, maintaining and promoting homeostasis, differentiation, development and survival, and preventing nutritional, metabolic, and infection-mediated stresses [73,74]. Autophagy is described by the presence of a large number of autophagosomes with cytoplasmic content [75]. Both types of cell death (apoptosis and autophagy) do not generate an inflammatory response, since the cytoplasmic membrane is conserved until the cellular debris are eliminated by neighborhood or specialized organelles. The cell death pathway caused by necrosis is considered an accidental, unprogrammed event that occurs under total ATP depletion, resulting from external stimuli such as extreme physical–chemical stress, heat, osmotic shock, mechanical stress, freezing, thawing, and high concentrations of hydrogen peroxide [76]. Necrotic cell death is morphologically characterized by generalized swelling of cell membranes, often accompanied by some condensation of nuclear chromatin, rupture of plasma membrane and an irregular DNA degradation pattern. Cytoplasmic membranes and membranous organelles dilate, and this increased swelling causes the breakdown of the plasma membrane, which releases the cytoplasmic contents into the extracellular space. The release of the intracellular contents, including proteins and nucleic acids, leads to massive cellular damage, affecting neighboring cells, which triggers inflammatory and autoimmune reactions. Necrosis takes place in the absence of phagocytosis, and its final phase is characterized by the loss of the integrity of the cellular membrane [77]. Necrosis is much more inflammatory than apoptosis or autophagy.

**Figure 18:**
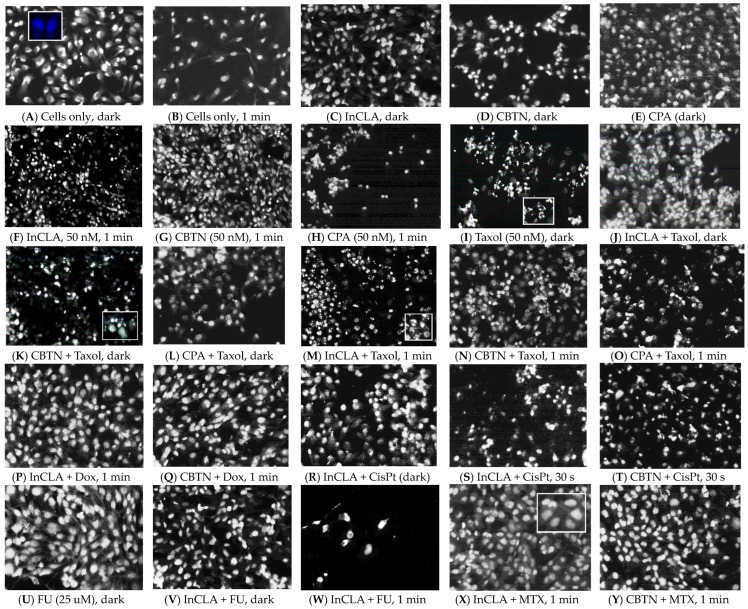
Fluorescence microscopy images of fixed human triple-negative breast cancer (TNBC) BT-549 cells. Grayscale images of cells stained with Hoechst 33258. Morphology of untreated unirradiated cells, sham control (**A**), illuminated cells (**B**), then of cells treated with: PSs (InCLA, CBTN, and CPA) and taxol in the dark (**C**–**E**); PSs, followed by 1 min light exposure (**F**–**H**); taxol unirradiated (**I**); PSs and taxol in the dark (**J**–**L**); PSs co-treated with taxol, then light-exposed (**M**–**O**); PSs co-treated then doxorubicin, irradiated (**P**,**Q**); PSs and cisplatin, dark (**R**); PSs and cisplatin, irradiated (**S**,**T**); fluorouracil, dark (**U**); InCLA with fluorouracil in the dark (**V**), and light-exposed (**W**); and PSs with methotrexate with light (**X**,**Y**). Inserts indicate enlarged view. Shrunk cells and chromatin condensation are evident upon treatment with taxol (**I**), on irradiated cells co-treated with PSs and taxol (**M**–**O**), and with cisplatin (**S**,**T**). Light dose in 1 min = 0.96 J cm^−2^; in 30 s = 0.48 J cm^−2^. Concentrations: PSs = 50 nM; taxol = 50 nM; doxorubicin = 500 nM; cisplatin = 25 μM; fluorouracil = 25 μM; and, methotrexate = 500 nM.

To determine the preferred cell death pathway for the binary chemophotodynamic therapy in this study, tracking the hallmarks of apoptosis or necrosis was accomplished using a nuclear stain to monitor the chromatin condensation and cellular contraction [78]. Fluorescence microscopy images of TNBC cells stained with Hoechst 33258 nuclear stain are shown in Figure 18. The TNBC cells were spindle-shaped and nearly-round, with intact cytoplasm, diffused chromatin, distinct cellular membrane, and large oval nuclei staining dark blue with the nuclear stain (Figure 18A,B). The PS-treated cells in the dark (Figure 18C–E) exhibited morphology similar to the untreated and irradiated sham control (Figure 18A,B). The InCLA- and CPA-treated cells clearly showed reduced cellular volume and nuclear density, but not as obvious as in the CBTN-treated cells at a 50 nM concentration for 24 h treatment followed by light exposure (Figure 18F–H). For the taxol-treated (Figure 18I) cells in the dark, and cells with PSs (InCLA, CBTN and CPA) co-treated with taxol also in the dark (Figure 18J–L), there was some evidence of cell shrinkage and chromatin fragmentation, showing apoptosis as the preferred pathway. Without light, only the effect of taxol on cell morphology was seen. However, upon light irradiation, the effect of combination treatment consisting of PS and taxol (Figure 18M–O) was more pronounced than with taxol treatment alone, in which more enhanced evidence of the morphological characteristics of the apoptotic pathway was observed. No significant difference was observed between the sham control cells and cells subjected to combination treatment with InCLA (50 nM) or CBTN (50 nM) with doxorubicin (500 nM) (Figure 18P,Q). With combined cisplatin and InCLA in the dark, very few shrunk cells could be seen, which was not significant (Figure 18R). However, when exposed to light for 30 s (light dose 0.48 J/cm^2^), a significant number of shrunk cells and chromatin condensation could be seen (Figure 18S,T) for the InCLA- or CBTN- and cisplatin-cotreated cells. The cells treated with fluorouracil at 25 μM in the dark did not affect the cellular morphology (Figure 18U), as well as with InCLA with fluorouracil (Figure 18V). However, upon light irradiation, an extremely reduced cell density was apparent (Figure 18W). Very few cells that exhibited chromatin condensation or any morphological changes indicative of apoptosis for cells treated with InCLA or CBTN co-treated with methotrexate followed by irradiation were detected (Figure 18X,Y).

The experimental results obtained using the fluorescence microscopy technique indicated that the preferred mode of cell death was apoptosis when our synthesized PSs (InCLA, CBTN, and CPA) were co-treated with taxol or cisplatin. No evidence of cell swelling suggesting necrosis was observed. The combination of PSs with fluorouracil showed a loss of cell viability, as indicated by the reduced cell density, possibly due to the loss of cell adhesion and contact with neighboring cells, but apoptosis seemed to be somewhat preferred over necrosis. Binary treatment using our PSs with doxorubicin or methotrexate did not show conclusive evidence of any cell death mechanism.

#### 2.3.2. Transmission Electron Microscopy Studies

Apoptosis, necrosis, and autophagy are now recognized as the most common mechanisms of cell deletions characterized by the peculiar morphology of a physiologically occurring cell death. Transmission electron microscopy (TEM) provides a detailed depiction of these phenomena and, due to its high resolution, is one of the most powerful morphological techniques used to visualize the inner cellular and organelle ultrastructural alterations under physiological and pathological conditions [79]. Histopathology can recognize differences in these three pathways [80].

**Figure 19:**
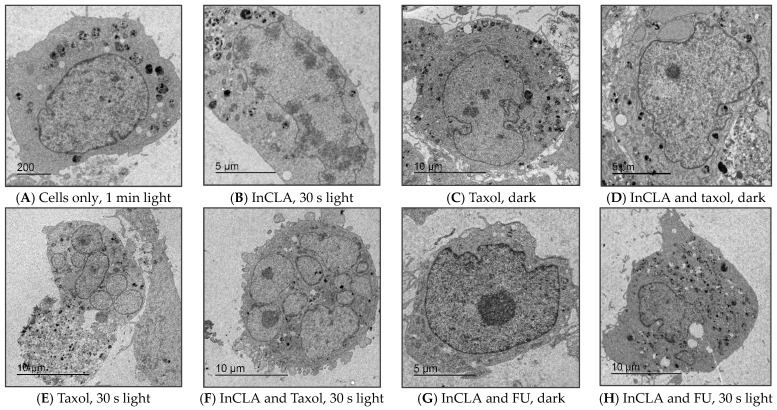
Ultrastructure TEM images of human triple-negative breast cancer (TNBC) BT-549 cells: untreated irradiated (**A**); PS-treated with InCLA and irradiated (**B**); taxol-treated in the dark (**C**); co-treatment with InCLA and taxol unirradiated (**D**); taxol-treated only and irradiated (**E**); co-treatment with InCLA and taxol, followed by light treatment (**F**); and, co-treatment with InCLA and FU (fluorouracil) in the dark (**G**), and with light treatment (**H**). Irregularly shaped nuclear membrane is apparent in treated cells in the dark or in the presence of light. Light dose in 30 s = 0.48 J cm^−2^. Concentrations: PSs = 50 nM; taxol = 50 nM; and, fluorouracil = 25 μM.

Transmission electron micrographs of TNBC cells clearly differentiated distinct cellular compartments, particularly of nuclear components and the plasma membrane, during treatment, as shown in Figure 19. A typical normal organelle morphology with smooth-contoured cellular plasma membrane was depicted in the TNBC cells irradiated with light without treatment with PS or a chemotherapeutic agent (Figure 19A). Chromatin showed an abnormal pattern, with an elongated nuclear envelope and numerous clumps of shredded DNA around the inner nuclear membrane in the InCLA-treated and irradiated cells (Figure 19B), indicative of the early phase of apoptosis. A lower light dose of 0.48 J/cm^2^ was applied to monitor the modifications that cells undergo during PDT treatment. The irregularly shaped nuclear envelope in the taxol-treated and in the InCLA co-treated cells in the dark, as shown in Figure 19C–D, also demonstrated cellular changes upon treatment. The effect of taxol alone without light was evident in the loss of nuclear circularity and formation of chromatin condensation. When the taxol-treated (Figure 19E) and PS co-treated (Figure 19F) TNBC cells were illuminated, smaller nuclei in multi-nucleated cells, indicating chromosomal condensation, were observed. Multinucleation is recognized as histopathological evidence of cells that are compromised, characterized by a smaller nuclear size and smaller nuclear circularity, and are associated with certain diseases [81,82]. The cytoplasm in the taxol-treated cells (Figure 19E) had split and fragmented, with many atypical vesicular components in the cytosolic compartment. Several ultrastructural features typical of apoptotic cell death were evident in the TNBC cells co-treated with InCLA and taxol in the presence of light (Figure 19F), such as cellular surface blebbing and overlapping disintegrated nuclear pores. In the cells co-treated with InCLA and fluorouracil in the dark (Figure 19G), the chromatin was abnormally condensed into a large central nuclear clump (stained dark), with chromatin marginalization and irregularly-shaped cellular membrane depicting the timeline during the early phase of the apoptotic pathway. In the presence of light (Figure 19H), large vacuoles and vesicles in the cytoplasm were observed.

Our representative TEM images confirmed our fluorescence microscopy results that apoptosis seemed to be the preferred mode of cell death during chemophotodynamic treatment. However, our TEM data appear to suggest that mitotic catastrophe and some autophagy also occurred, similar to another study conducted on the effect of paclitaxel in a human gastric adenocarcinoma AGS cell line, in which apoptosis, autophagy and mitotic catastrophe were triggered by paclitaxel [83]. Aside from the most common mode of cell death mechanistic pathways, such as apoptosis (manifested by volume reduction of nucleus and cytoplasmic or cell shrinkage), necrosis (cytoplasmic or mitochondrial swelling and plasma membrane rupture), and autophagy (accumulation of cytosolic vacuoles and membranes), mitotic catastrophe is also a cell death mechanism triggered by aberrant or dysregulated mitosis. The morphological markers of mitotic catastrophe are micronucleation or multinucleation, in which multinucleated cells are formed from the clusters of mis-segregated and uncondensed chromosomes [84]. Hence, multiple cell death mechanisms were, to some degree, activated during combination therapy in this study.

## 3. Materials and Methods

**General.** Human mammary epithelial carcinoma cell line purchased from the American Type Culture Collection BT-549 (ATCC HTB-122) was cultured according to the ATCC protocol (ATCC, Manassas, VA, USA). Briefly, BT-549 cells were grown in RPMI 1640 containing 0.023 IU/mL insulin and supplemented with 10% fetal bovine serum (FBS). Cells were grown to 80–90% confluence in 75 cm^2^ Corning culture flasks (Fisher, Waltham, MA, USA) for 4–5 days in a Fisher Scientific Isotemp humidified incubator (Fisher, Waltham, MA, USA) with 5% CO_2_ at 37 °C. During the incubation period, the growth media were changed once for fresh pre-warmed media (pH 7.2). To harvest the cells, the old growth media were aspirated out and 3 mL of 0.25% Thermo Sci Hyclone trypsin solution (Fisher, Waltham, MA, USA) was added. The cells were incubated for 5 min and the cell pellet after centrifugation was resuspended in 3 mL media, broken up gently; then, 1 mL of suspended cells was transferred into a new T75 cell culture flask containing pre-warmed media (20 mL) for further culturing.

### 3.1. Cell Cytotoxicity Assay

Cells were grown to confluence in a 96-well plate (9 × 10^3^ cells/well) and co-treated for 24 h with our synthesized photosensitizers and chemotherapeutic agents of varying concentrations (ranging from 5 nM to 50 μM) in growth media from a stock solution of 10 mM in DMSO or dimethyl sulfoxide(Fisher, Waltham, MA, USA). After 24 h of treatment, the old growth media containing the PS or compounds were aspirated out and replaced with fresh media. The plates were then positioned below a non-coherent LumaCare LC-122 650 nm light source (LumaCare, Newport Beach, CA, USA) for 1 min at an energy fluence rate of 0.96 J/cm^2^ and a power of 16 mW/cm^2^, measured using a Newport optical power meter Model 840 (Newport, Bozeman, MT, USA). Unirradiated cells served as control samples. The following day, the cells were washed with pre-warmed PBS, and (3-[4,5-dimethyl-thiazol-2-yl]-2.5-diphenyltetrazolium bromide) or MTT (Sigma, St. Louis, MO, USA) with a concentration of 0.3 mg/mL) in PBS was added to each well. The samples were allowed to incubate for additional 2 h, after which dark blue crystals formed. DMSO was added to each well and the plates were shaken at room temperature for 1 h to dissolve the purplish–blue formazan crystals. The absorbance values at 570 nm were measured on a BioRad 550 microplate reader (BioRad, Hercules, CA, USA). Cell survival was calculated based on the absorbance of the untreated cells alone (as a control) and was directly proportional to the number of viable cells in the culture. The results were reported as the mean ± SD of triplicate measurements in three separate experiments.

### 3.2. Dose–Effect and Drug Combination Analysis

The drug combination index was calculated based on the Chou–Talalay method available online as freeware CompuSyn Version 1.0 (https://www.combosyn.com) [70]. The program provided quantitation of the synergism and antagonism in the drug combinations. The combination index (CI) was then determined based on the algorithm using experimental data derived from the cell cytotoxicity assay of the PSs alone and PSs combined with chemotherapeutic agents. The specific concentrations used for single dose of PS and chemotherapeutic agent, and the combinations, were entered. The CI index calculated was matched using Table 4.1 from the CompuSyn User’s Guide, with the CI indices ranging from <0.1 to 1.00 to >10 being described as very strongly synergistic to additive to very strongly antagonistic, respectively.

### 3.3. Statistical Analysis

Statistical analysis of the effect of the combination treatment was conducted independently, with separate and appropriate controls for the different combination treatments of PS and chemotherapeutic drugs. The results were represented as the mean ± standard error. Replicate measurements were performed three times, with the assays run in triplicate. Statistical significance was defined as a *p* (probability) value less than 0.05, which was evaluated using the two-tailed *t*-test (paired samples for means) available in the Data Analysis toolbox in Excel.

### 3.4. Fluorescence Microscopy

Cells (1 mL aliquots) obtained from a diluted cell suspension were seeded into each well (1.7 cm^2^, 8 × 10^3^ cells/well) of a 4-well BD Biosciences culture slide (Fisher, Waltham, MA, USA) and grown to confluence in 5% CO_2_ at 37 °C for 3–4 days for attachment to the substratum. After aspirating the old growth media, 1 mL of the photosensitizer and chemotherapeutic agents (at appropriate concentrations) in fresh pre-warmed media at 37 °C was added to each well. After compound treatment for 24 h, the cells were washed twice with 1 mL fresh growth media and then irradiated with light using the LumaCare LC-122, as described above. The cells were stained in the dark with Hoechst 33258 (Fisher, Waltham, MA, USA) in pre-warmed media for 10 min at 37 °C, washed twice with filtered PBS, and then fixed with filtered paraformaldehyde for 15 min in the incubator. After thorough liquid aspiration, the wells were removed and allowed to air dry in the dark for 1 h. The slides were protected with coverslips, whose edges were sealed using a clear fast-drying nail polish and allowed to dry at room temperature in the dark for 30 min. Images were recorded using fluorescence microscopy (DAPI for Hoechst 350–390 nm excitation and 460–490 nm emission filters) using an upright fluorescence microscope with Retiga imaging 2000R (Nikon Optiphot-2, 20× and 40×) and the image-processing Nikon NIS-Elements V4.0 Qimaging software (Nikon Instruments, Melville, NY, USA).

### 3.5. Transmission Electron Microscopy

Cells were cultured to confluence in a Petri dish (50 cm in diameter), treated for 24 h with 500 nM of PS, and then irradiated for 2 min as above. After photosensitization 24 h later, the cells were scraped gently in the dark, fixed in 2.5% glutaraldehyde in 0.1 M sodium cacodylate buffer, then post-fixed with 1% osmium tetroxide (containing 0.8% ferricyanide), treated with 2% aqueous uranyl acetate, and subsequently dehydrated in gradient concentrations (50–100%) of varying ethanol:water mixtures. The resulting pellets were embedded in resin and cut with an ultramicrotome to a 70 nm thickness. They were then viewed using a Tecnai T20 transmission electron microscope.

## 4. Conclusions

The experimental results presented herein proved that our synthesized chlorin–vitamin conjugated photosensitizers (InCLA, CPA and CBTN) can be combined with taxol, producing the best chemophotodynamic efficacy in vitro against a TNBC cell line in the nanomolar concentration range, followed by other combinations with cisplatin, fluorouracil and methotrexate but at a higher concentration of the chemotherapeutic drug. The study presented here demonstrated that the ternary combination of PDT (a binary therapy) and chemotherapy is a potential alternative treatment for TNBC patients. Chemotherapy combined with PDT (specifically, InCLA-taxol) resulted in a synergistic anti-tumor effect and reduced the dosage of both the chemotherapeutic drug and the photosensitizer in the nanomolar concentration range. Further studies are warranted to elucidate and fully understand the underlying mechanisms involved during binary therapy and the effect of the molecular structures of the PSs on their photodynamic efficacy.

## Figures and Tables

**Table 1:** Cell inhibition (%) of TNBC cells treated with photosensitizers (100 nM) and chemotherapeutic drugs for 24 h, followed by light irradiation (light dose of 0.96 J cm^−2^). Chemotherapeutic drugs: taxol (50 nM), doxorubicin (500 nM), cisplatin (25 μM), fluorouracil (25 μM), and methotrexate (500 nM).

Photosensitizers	Taxol	Doxorubicin	Cisplatin	Fluorouracil	Methotrexate
MePheo	55	36	39	34	40
CBTN	65	64	53	64	42
CBX	48	30	0	6	42
CPA	63	73	87	43	33
CLA	45	33	65	35	43
InCBTN	45	25	0	13	33
InCBX	45	41	16	9	42
InCPA	51	27	0	25	30
InCLA	91	91	90	92	61

**Table 2:** Combination index (CI) values of binary therapy (selected PSs and chemotherapeutic drugs) and the corresponding synergism vs. additive vs. antagonism effects. CI = 1 (additive); CI < 1 (synergism); CI > 1 (antagonism). VSS (< 0.1), very strong synergism; SS (0.1–0.3), strong synergism; S (0.3–0.7), synergism; MS (0.7–0.85), moderate synergism; StS (0.85–0.90), slight synergism; NA (0.9–1.10), nearly additive; StA (1.10–1.20), slight antagonism; MA (1.20–1.45), moderate antagonism; A (1.45–3.3), antagonism; SA (3.3–10), strong antagonism; VSA (>10), very strong antagonism. Concentrations: PSs = 50 nM; taxol = 50 nM; doxorubicin = 500 nM; cisplatin = 25 μM; fluorouracil = 25 μM; methotrexate = 500 nM.

PSs 50 nM	Taxol 50 nM	Doxorubicin 500 nM	Cisplatin 25 μM	Fluorouracil 25 μM	Methotrexate 500 nM
MePheo	13.27 (VSA)	6.37 (SA)	6.10 (SA)	17.96 (VSA)	7.79 (SA)
CBTN	0.74 (MS)	2.44 (A)	0.99 (NA)	0.48 (S)	1.57 (A)
CPA	0.41 (S)	1.52 (A)	1.30 (MA)	0.45 (S)	0.50 (S)
InCLA	0.25 (SS)	1.91 (MA)	1.09 (NA)	0.94 (NA)	1.21 (MA)

## Data Availability

Data are contained within the article.

## Supplementary Materials

The following supporting information can be downloaded at: https://www.mdpi.com/article/10.3390/ph17050576/s1, Figure S1: Cell survival assay of TNBC cells treated with photosensitizers at 100 nM and at 100 nM co-treated with 25 μM cisplatin in the dark (A), in the presence of light (B), and compared to cisplatin-only treated cells; Figure S2: Cell survival assay of TNBC cells treated with photosensitizers at 100 nM and at 100 nM co-treated with 25 μM fluorouracil in the dark (A), in the presence of light (B), and compared to fluorouracil-only treated cells. Figure S3. Cell survival assay of TNBC cells treated with photosensitizers at 100 nM and at 100 nM co-treated with 500 nM methotrexate in the dark (A), in the presence of light (B), and compared to methotrexate-only treated cells. Table S1: Toxicology Data of the Chemotherapeutic Drugs Used in this Study. Table S2: Acute Oral Toxicity Categories and Classification Criteria.

## References

[1] Siegel R., Giaquinto A.N., Jemal A. (2024). Cancer Statistics 2024. CA Cancer J. Clin..

[2] Łukasiewicz S., Czeczelewski M., Forma A., Baj J., Sitarz R., Stanisławek A. (2021). Breast Cancer—Epidemiology, Risk Factors, Classification, Prognostic Markers, and Current Treatment Strategies—An Updated Review. Cancers.

[3] Schick J., Ritchie R.P., Restini C. (2021). Breast Cancer Therapeutics and Biomarkers: Past, Present, and Future Approaches. Breast Cancer Basic Clin. Res..

[4] Romero Lagunes M.L., Pezo R.C. (2021). A narrative of chemotherapy in advanced triple negative breast cancer. Precis. Cancer Med..

[5] Claessens A.K.M., Ibragimova K.I.E., Geurts S.M.E., Bos M.E.M.M., Erdkamp F.L.G., Tjan-Heijnen V.C.G. (2020). The role of chemotherapy in treatment of advanced breast cancer: An overview for clinical practice. Crit. Rev. Oncol. Hematol..

[6] Landry I., Sumbly V., Vest M. (2022). Advancements in the Treatment of Triple-Negative Breast Cancer: A Narrative Review of the Literature. Cureus.

[7] Won K.A., Won K.A. (2020). Triple-negative breast cancer therapy: Current and future perspectives (Review). Int. J. Oncol..

[8] Adel N.G. (2021). Current Treatment Landscape and Emerging Therapies for Metastatic Triple-Negative Breast Cancer. Am. J. Manag. Care.

[9] Almansour N.M. (2022). Triple-Negative Breast Cancer: A Brief Review About Epidemiology, Risk Factors, Signaling Pathways, Treatment and Role of Artificial Intelligence. Front. Mol. Biosci..

[10] Bianchini G., De Angelis C., Licata L., Gianni L. (2022). Treatment landscape of triple-negative breast cancer—Expanded options, evolving needs. Nat. Rev. Clin. Oncol..

[11] Zong Y., Pegram M. (2021). Research advances and new challenges in overcoming triple-negative breast cancer. Cancer Drug Resist..

[12] Shen M., Pan H., Chen Y., Xu Y.H., Yang W., Wu Z. (2020). A review of current progress in triple-negative breast cancer therapy. Open Med..

[13] Robson M., Im S.A., Senkus E., Xu B., Domchek S.M., Masuda N., Delaloge S., Li W., Tung N., Armstrong A. (2017). Olaparib for Metastatic Breast Cancer in Patients with a Germline BRCA Mutation. N. Engl. J. Med..

[14] Robson M.E., Tung N., Conte P., Im S.A., Senkus E., Xu B., Masuda N., Delaloge S., Li W., Armstrong A. (2019). OlympiAD final overall survival and tolerability results: Olaparib versus chemotherapy treatment of physician’s choice in patients with a germline BRCA mutation and HER2-negative metastatic breast cancer. Ann. Oncol..

[15] Schmid P., Adams S., Rugo H.S., Schneeweiss A., Barrios C.H., Iwata H., Diéras V., Hegg R., Im S.A., Shaw Wright G. (2018). Atezolizumab and Nab-Paclitaxel in Advanced Triple-Negative Breast Cancer. N. Engl. J. Med..

[16] Algorri J.F., Ochoa M., Roldán-Varona P., Rodríguez-Cobo L., López-Higuera J.M. (2021). Photodynamic Therapy: A Compendium of Latest Reviews. Cancers.

[17] Niculescu A.-G., Grumezescu A.M. (2021). Photodynamic Therapy—An Up-to-Date Review. Appl. Sci..

[18] Correia J.H., Rodrigues J.A., Pimenta S., Dong T., Yang Z. (2021). Photodynamic Therapy Review: Principles, Photosensitizers, Applications, and Future Directions. Pharmaceutics.

[19] Gunaydin G., Gedik M.E., Ayan S. (2021). Photodynamic Therapy for the Treatment and Diagnosis of Cancer-A Review of the Current Clinical Status. Front. Chem..

[20] Dos Santos A.F., de Almeida D.R.Q., Terra L.F., Baptista M.S., Labriola L. (2019). Photodynamic therapy in cancer treatment—An update review. J. Cancer Metastasis Treat..

[21] Triesscheijn M., Baas P., Schellens J.H., Stewart F.A. (2006). Photodynamic therapy in oncology. Oncologist.

[22] Isaac-Lam M.F., Hammonds D.M. (2019). Synthesis and Photodynamic Activity of Vitamin-Chlorin Conjugates at the Nanomolar Concentrations Against Prostate Cancer Cells. ACS Omega.

[23] Isaac-Lam M.F., Mee A.D. (2019). Photodynamic Activity of Chlorin-Vitamin Conjugates at the Nanomolar Concentrations Against Triple-Negative Breast Cancer Cells. ACS Omega.

[24] Isaac-Lam M.F., Hammonds D.M. (2017). Biotinylated Chlorin and Its Zinc and Indium Complexes: Synthesis and In Vitro Biological Evaluation for Photodynamic Therapy. Pharmaceuticals.

[25] Aniogo E.C., Plackal Adimuriyil George B., Abrahamse H. (2019). The role of photodynamic therapy on multidrug resistant breast cancer. Cancer Cell Int..

[26] Isaac-Lam M.F. (2021). Chlorin-Vitamin Conjugates for Triple-Negative Breast Cancer. U.S. Patent.

[27] Isaac-Lam M.F. (2020). Chlorin-Vitamin Conjugates as Cancer Therapeutics. U.S. Patent.

[28] Weaver B.A. (2014). How Taxol/paclitaxel kills cancer cells. Mol. Biol. Cell.

[29] Abu Samaan T.M., Samec M., Liskova A., Kubatka P., Büsselberg D. (2019). Paclitaxel’s Mechanistic and Clinical Effects on Breast Cancer. Biomolecules.

[30] Asghari F., Haghnavaz N., Shanehbandi D., Khaze V., Baradaran B., Kazemi T. (2018). Differential altered expression of let-7a and miR-205 tumor-suppressor miRNAs in different subtypes of breast cancer under treatment with Taxol. Adv. Clin. Exp. Med..

[31] Smith E.R., Xu X.-X. (2021). Breaking malignant nuclei as a non-mitotic mechanism of taxol/paclitaxel. J. Cancer Biol..

[32] Xu J., Zheng Q., Cheng X., Hu S., Zhang C., Zhou X., Sun P., Wang W., Su Z., Zou T. (2021). Chemo-photodynamic therapy with light-triggered disassembly of theranostic nanoplatform in combination with checkpoint blockade for immunotherapy of hepatocellular carcinoma. J. Nanobiotechnol..

[33] Thapa P., Li M., Bio M., Rajaputra P., Nkepang G., Sun Y., Woo S., You Y. (2016). Far-Red Light-Activatable Prodrug of Paclitaxel for the Combined Effects of Photodynamic Therapy and Site-Specific Paclitaxel Chemotherapy. J. Med. Chem..

[34] Baglo Y., Sorrin A.J., Liang B.J., Huang H.C. (2020). Harnessing the Potential Synergistic Interplay Between Photosensitizer Dark Toxicity and Chemotherapy. Photochem. Photobiol..

[35] Pommier Y., Leo E., Zhang H.-L., Marchand C. (2010). DNA Topoisomerases and Their Poisoning by Anticancer and Antibacterial Drugs. Chem. Biol..

[36] Kim S.Y., Kim S.J., Kim B.J., Rah S.Y., Chung S.M., Im M.J., Kim U.H. (2006). Doxorubicin-induced reactive oxygen species generation and intracellular Ca2+ increase are reciprocally modulated in rat cardiomyocytes. Exp. Mol. Med..

[37] Yang F., Kemp C.J., Henikoff S. (2013). Doxorubicin enhances nucleosome turnover around promoters. Curr. Biol..

[38] Cacaccio J.C., Durrani F.A., Missert J.R., Pandey R.K. (2022). Photodynamic Therapy in Combination with Doxorubicin Is Superior to Monotherapy for the Treatment of Lung Cancer. Biomedicines.

[39] Aniogo E.C., George B.P.A., Abrahamse H. (2017). In vitro combined effect of Doxorubicin and sulfonated zinc Phthalocyanine-mediated photodynamic therapy on MCF-7 breast cancer cells. Tumour Biol..

[40] Lanks K.W., Gao J.P., Sharma T. (1994). Photodynamic enhancement of doxorubicin cytotoxicity. Cancer Chemother. Pharmacol..

[41] Egger S.J., Chan M.M., Luo Q., Wilcken N. (2020). Platinum-containing regimens for triple-negative metastatic breast cancer. Cochrane Database Syst. Rev..

[42] Dasari S., Tchounwou P.B. (2014). Cisplatin in cancer therapy: Molecular mechanisms of action. Eur. J. Pharmacol..

[43] Tchounwou P.B., Dasari S., Noubissi F.K., Ray P., Kumar S. (2021). Advances in Our Understanding of the Molecular Mechanisms of Action of Cisplatin in Cancer Therapy. J. Exp. Pharmacol..

[44] Aldossary S.A. (2019). Review on Pharmacology of Cisplatin: Clinical Use, Toxicity and Mechanism of Resistance of Cisplatin. Biomed. Pharmacol. J..

[45] Cheng Y.S., Peng Y.B., Yao M., Teng J.P., Ni D., Zhu Z.J., Zhuang B.F., Yang Z.Y. (2017). Cisplatin and photodynamic therapy exert synergistic inhibitory effects on small-cell lung cancer cell viability and xenograft tumor growth. Biochem. Biophys. Res. Commun..

[46] Javani Jouni F., Abdollahi V., Zadehmodarres S., Abbasinia H., Asnaashari M., Zafari J. (2022). Combination of cisplatin treatment and photodynamic therapy attenuates cisplatin-induced cell toxicity in A2780 and A2780-CP cervical cancer cell lines. Lasers Med. Sci..

[47] Ahn T.G., Jung J.M., Lee E.J., Choi J.H. (2019). Effects of cisplatin on photosensitizer-mediated photodynamic therapy in breast tumor-bearing nude mice. Obstet. Gynecol. Sci..

[48] Rizvi I., Celli J.P., Evans C.L., Abu-Yousif A.O., Muzikansky A., Pogue B.W., Finkelstein D., Hasan T. (2010). Synergistic enhancement of carboplatin efficacy with photodynamic therapy in a three-dimensional model for micrometastatic ovarian cancer. Cancer Res..

[49] Robertson J., Barr R., Shulman L.N., Forte G.B., Magrini N. (2016). Essential medicines for cancer: WHO recommendations and national priorities. Bull. World Health Organ..

[50] Rutman R.J., Cantarow A., Paschkis K.E. (1954). Studies in 2-acetylaminofluorene carcinogenesis. III. The utilization of uracil-2-C14 by preneoplastic rat liver and rat hepatoma. Cancer Res..

[51] Sommer H., Santi D.V. (1974). Purification and amino acid analysis of an active site peptide from thymidylate synthetase containing covalently bound 5-fluoro-2’-deoxyuridylate and methylenetetrahydrofolate. Biochem. Biophys. Res. Commun..

[52] Haritha N.H., Nawab A., Vijayakurup V., Anto N.P., Liju V.B., Alex V.V., Amrutha A.N., Aiswarya S.U., Swetha M., Vinod B.S. (2021). Targeting Thymidylate Synthase Enhances the Chemosensitivity of Triple-Negative Breast Cancer Towards 5-FU-Based Combinatorial Therapy. Front. Oncol..

[53] Chalabi-Dchar M., Fenouil T., Machon C., Vincent A., Catez F., Marcel V., Mertani H.C., Saurin J.-C., Bouvet P., Guitton J. (2021). A novel view on an old drug, 5-fluorouracil: An unexpected RNA modifier with intriguing impact on cancer cell fate. NAR Cancer.

[54] Su P., Ahmad B., Zou K., Zou L. (2020). β-Elemene Enhances the Chemotherapeutic Effect of 5-Fluorouracil in Triple-Negative Breast Cancer via PI3K/AKT, RAF-MEK-ErK, and NF-κB Signaling Pathways. OncoTargets Ther..

[55] Ponce-Cusi R., Calaf G.M. (2016). Apoptotic activity of 5-fluorouracil in breast cancer cells transformed by low doses of ionizing α-particle radiation. Int. J. Oncol..

[56] Maytin E.V., Anand S., Riha M., Lohser S., Tellez A., Ishak R., Karpinski L., Sot J., Hu B., Denisyuk A. (2018). 5-Fluorouracil Enhances Protoporphyrin IX Accumulation and Lesion Clearance during Photodynamic Therapy of Actinic Keratoses: A Mechanism-Based Clinical Trial. Clin. Cancer Res..

[57] Zhao B., Li L., Lei Q., Guan K.L. (2010). The Hippo-YAP pathway in organ size control and tumorigenesis: An updated version. Genes Dev..

[58] Zhou Y., Wang Y., Zhou W., Chen T., Wu Q., Chutturghoon V.K., Lin B., Geng L., Yang Z., Zhou L. (2019). YAP promotes multi-drug resistance and inhibits autophagy-related cell death in hepatocellular carcinoma via the RAC1-ROS-mTOR pathway. Cancer Cell Int..

[59] Chan E.S., Cronstein B.N. (2013). Mechanisms of action of methotrexate. Bull. Hosp. Jt. Dis..

[60] Allegra C.J., Chabner B.A., Drake J.C., Lutz R., Rodbard D., Jolivet J. (1985). Enhanced inhibition of thymidylate synthase by methotrexate polyglutamates. J. Biol. Chem..

[61] Baggott J.E., Vaughn W.H., Hudson B.B. (1986). Inhibition of 5-aminoimidazole-4-carboxamide ribotide transformylase, adenosine deaminase and 5’-adenylate deaminase by polyglutamates of methotrexate and oxidized folates and by 5-aminoimidazole-4-carboxamide riboside and ribotide. Biochem. J..

[62] Fairbanks L.D., Rückermann K., Qiu Y., Hawrylowicz C.M., Richards D.F., Swaminathan R., Kirschbaum B., Simmonds H.A. (1999). Methotrexate Inhibits the First Committed Step of Purine Biosynthesis in Mitogen-Stimulated Human T-lymphocytes: A Metabolic Basis for Efficacy in Rheumatoid Arthritis?. Biochem. J..

[63] Nogueira E., Sarria M.P., Azoia N.G., Antunes E., Loureiro A., Guimaraes D., Noro J., Rollett A., Guebitz G., Cavaco-Paulo A. (2018). Internalization of Methotrexate Conjugates by Folate Receptor-α. Biochemistry.

[64] Koźmiński P., Halik P.K., Chesori R., Gniazdowska E. (2020). Overview of Dual-Acting Drug Methotrexate in Different Neurological Diseases, Autoimmune Pathologies and Cancers. Int. J. Mol. Sci..

[65] Ali S., Muhammad S., Khurshid A., Ikram M., Maqsood M., Fisher C., Cathcart J., Lilge L. (2018). Effective phthalocyanines mediated photodynamic therapy with doxorubicin or methotrexate combination therapy at sub-micromolar concentrations in vitro. Photodiagn. Photodyn. Ther..

[66] Anand S., Honari G., Hasan T., Elson P., Maytin E.V. (2009). Low-dose methotrexate enhances aminolevulinate-based photodynamic therapy in skin carcinoma cells in vitro and in vivo. Clin. Cancer Res..

[67] Emran T.B., Shahriar A., Mahmud A.R., Rahman T., Abir M.H., Siddiquee M.F., Ahmed H., Rahman N., Nainu F., Wahyudin E. (2022). Multidrug Resistance in Cancer: Understanding Molecular Mechanisms, Immunoprevention and Therapeutic Approaches. Front. Oncol..

[68] Chou T.-C. (2010). Drug Combination Studies and Their Synergy Quantification Using the Chou-Talalay Method. Cancer Res..

[69] Elwakeel A., Soudan H., Eldoksh A., Shalaby M., Eldemellawy M., Ghareeb D., Abouseif M., Fayad A., Hassan M., Saeed H. (2019). Implementation of the Chou-Talalay method for studying the in vitro pharmacodynamic interactions of binary and ternary drug combinations on MDA-MB-231 triple negative breast cancer cells. Synergy.

[70] https://www.combosyn.com/.

[71] Carneiro B.A., El-Deiry W.S. (2020). Targeting apoptosis in cancer therapy. Nat. Rev. Clin. Oncol..

[72] Pfeffer C.M., Singh A.T.K. (2018). Apoptosis: A Target for Anticancer Therapy. Int. J. Mol. Sci..

[73] Aman Y., Schmauck-Medina T., Hansen MMorimoto R.I., Simon A.K., Bjedov I., Palikaras K., Simonsen A., Johansen T., Tavernarakis N., Rubinsztein D.C. (2021). Autophagy in healthy aging and disease. Nat. Aging.

[74] Ichimiya T., Yamakawa T., Hirano T., Yokoyama Y., Hayashi Y., Hirayama D., Wagatsuma K., Itoi T., Nakase H. (2020). Autophagy and Autophagy-Related Diseases: A Review. Int. J. Mol. Sci..

[75] Levine B., Klionsky D.J. (2004). Development by self-digestion: Molecular mechanisms and biological functions of autophagy. Dev. Cell.

[76] Escobar M.L., Echeverría O.M., Vázquez-Nin G.H. (2015). Necrosis as Programmed Cell Death. Cell Death—Autophagy, Apoptosis and Necrosis.

[77] Liu Z.G., Jiao D. (2019). Necroptosis, tumor necrosis and tumorigenesis. Cell Stress.

[78] Godwin W.C., Hoffmann G.F., Gray T.J., Hughes R.M. (2019). Imaging of morphological and biochemical hallmarks of apoptosis with optimized optogenetic tools. J. Biol. Chem..

[79] Rembiałkowska N., Dubińska-Magiera M., Sikora A., Szlasa W., Szewczyk A., Czapor-Irzabek H., Daczewska M., Saczko J., Kulbacka J. (2020). Doxorubicin Assisted by Microsecond Electroporation Promotes Irreparable Morphological Alternations in Sensitive and Resistant Human Breast Adenocarcinoma Cells. Appl. Sci..

[80] Ostańska E., Barnaś E., Bartusik-Aebisher D., Dynarowicz K., Szpunar M., Skręt-Magierło J., Aebisher D. (2022). Histopathological Analysis of the Effect of Photodynamic Action on Post-Chemotherapy Excised Breast Cancer Tissue. Medicina.

[81] Hart M., Adams S.D., Draviam V.M. (2021). Multinucleation associated DNA damage blocks proliferation in p53-compromised cells. Commun. Biol..

[82] Sugita S., Munechika R., Nakamura M. (2019). Multinucleation of Incubated Cells and Their Morphological Differences Compared to Mononuclear Cells. Micromachines.

[83] Khing T.M., Choi W.S., Kim D.M., Po W.W., Thein W., Shin C.Y., Sohn U.D. (2021). The effect of paclitaxel on apoptosis, autophagy and mitotic catastrophe in AGS cells. Sci. Rep..

[84] Imreh G., Helin Norberg H.V., Imreh S., Zhivotovsky B. (2011). Chromosomal breaks during mitotic catastrophe trigger γH2AX–ATM–p53-mediated apoptosis. J. Cell Sci..

