# Supplementary material for: Chlorin Conjugates in Photodynamic Chemotherapy for Triple-Negative Breast Cancer
Source: Pharmaceuticals (Basel). 2024 Apr 30;17(5):576. doi: 10.3390/ph17050576 (PMC11124301; doi:10.3390/ph17050576)
Supplement: Supplementary file 1 [file pharmaceuticals-17-00576-s001.zip › pharmaceuticals-2745130-supplementary.pdf]

Chlorin Conjugates in Photodynamic Chemotherapy for Triple-Negative Breast Cancer

Supplementary Material

Table S1. Toxicology Data of the Chemotherapeutic Drugs Used in this Study.

Chemotherapeutic Drug	Hazards from SDS Sheets	Toxicity[1a] Health Code	LD_{50} (literature)
Taxol	Cause skin irritation, serious eye damage, respiratory irritation, suspected of damaging fertility or the unborn child	2	32.5 mg/kg[2a]
Doxorubicin	Cause skin irritation, serious eye damage, respiratory irritation, may cause genetic defects, may cause cancer, suspected of damaging fertility, suspected of damaging the unborn child	4	570 mg/kg[2a]
Cisplatin	Fatal if swallowed, auses skin irritation, and serious eye damage, may cause respiratory irritation, may cause cancer, harmful in contact with skin or if inhaled	2	25.8 mg/kg[2b]
5-Fluorouracil	Toxic if swallowed, cause skin irritation, serious eye irritation, may cause respiratory irritation, suspected of causing genetic defects, suspected of damaging the unborn child, harmful in contact with skin or if inhaled	3	230 mg/kg[2c]
Methotrexate	Toxic if swallowed, cause skin irritation, serious eye irritation, may cause respiratory irritation, may cause genetic defects, may damage fertility, may damage the unborn child	3	135 mg/kg[2c]

[1a]Acute Oral Toxicity. Table VII.1.1. OSHA. https://www.osha.gov/sites/default/files/publications/OSHA3844.pdf. Last accessed 12/03/23.

Table S2. Acute Oral Toxicity Categories and Classification Criteria[1b]

Classification Criteria	Category 1	Category 2	Category 3	Category 4
Oral LD_{50}	≤ 5 mg/kg bodyweight	>5 and ≤ 50 mg/kg bodyweight	>50 and ≤ 300 mg/kg bodyweight	>300 and ≤ 2000 mg/kg bodyweight

[1b]Derived from Table VII.1.1. OSHA. https://www.osha.gov/sites/default/files/publications/OSHA3844.pdf. Last accessed 12/03/23.

LD_{50} = median lethal dose in rat (intraperitoneal)[2a], in mouse (oral)[2b], and in rat (oral)[2c] based on SDS (Safety Data Sheets):

Paclitaxel SDS: Last accessed 12/03/23.
https://www.fishersci.com/store/msds?partNumber=AC328420250&productDescription=PACLITAXEL+99%2B%25+25MGPACLITAXE&vendorId=VN00032119&countryCode=US&language=en

Doxorubicin SDS: Last accessed 12/03/23.
https://www.fishersci.com/store/msds?partNumber=AAJ64000MF&productDescription=DOXORUBICIN+HYDROCHLORIDE+50MG&vendorId=VN00024248&countryCode=US&language=en; https://cdn.pfizer.com/pfizercom/products/material_safety_data/PZ00059.pdf

Cisplatin SDS: Last accessed 12/03/23.
https://www.fishersci.com/store/msds?partNumber=AC193760050&countryCode=US&language=en

Fluorouracil SDS: Last accessed 12/03/23.
https://www.fishersci.com/store/msds?partNumber=AC228440250&productDescription=5-FLUOROURACIL%2C+99%25+25GR&vendorId=VN00032119&countryCode=US&language=en

Methotrexate SDS: Last accessed 12/03/23.
https://www.fishersci.com/store/msds?partNumber=AAJ6307503&productDescription=METHOTREXATE+1G&vendorId=VN00024248&countryCode=US&language=en

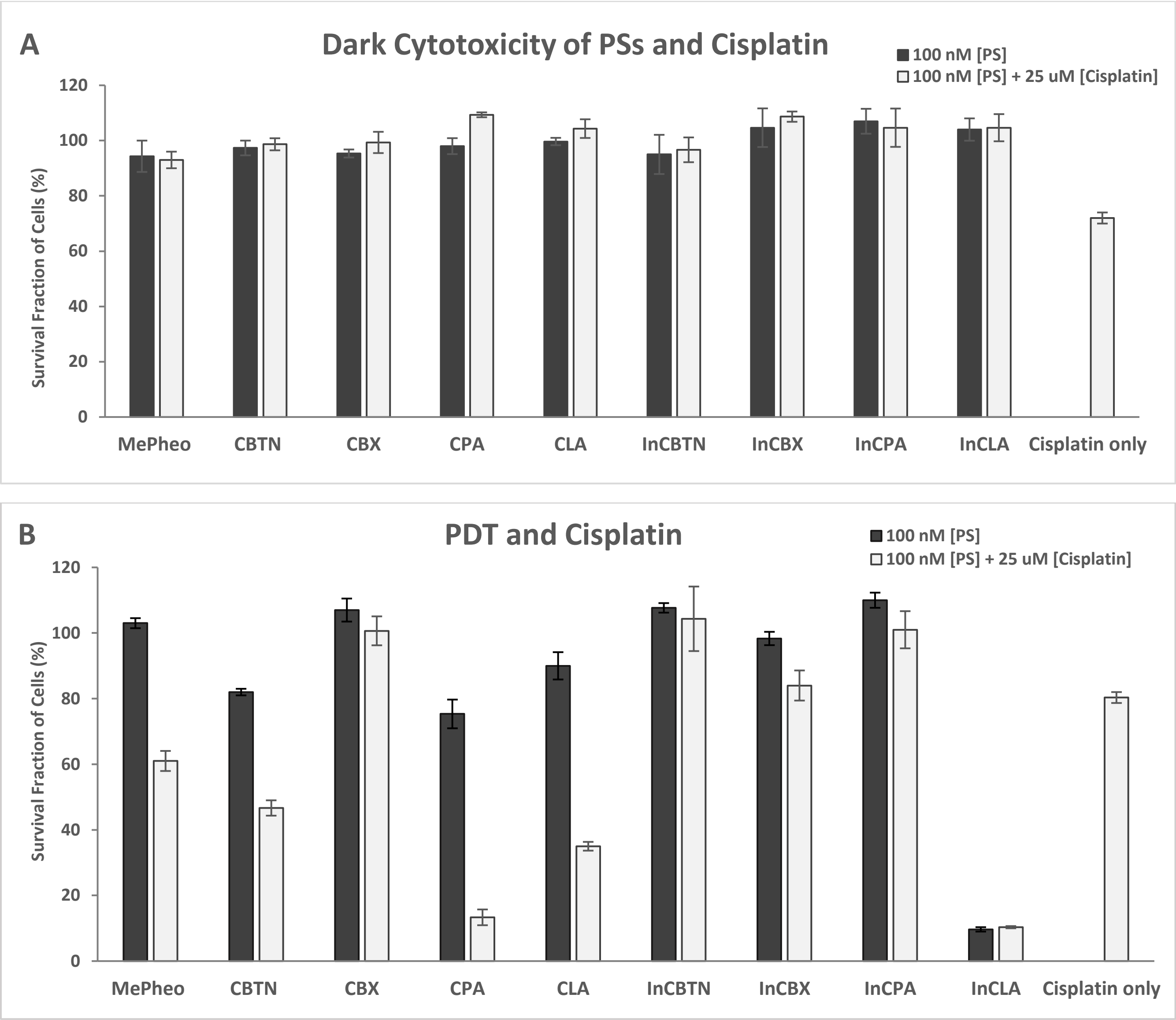

Figure S1. Cell survival assay of TNBC cells treated with photosensitizers at 100 nM and at 100 nM co-treated with 25 μM cisplatin in the dark (A), in the presence of light (B), and compared to cisplatin-only treated cells.

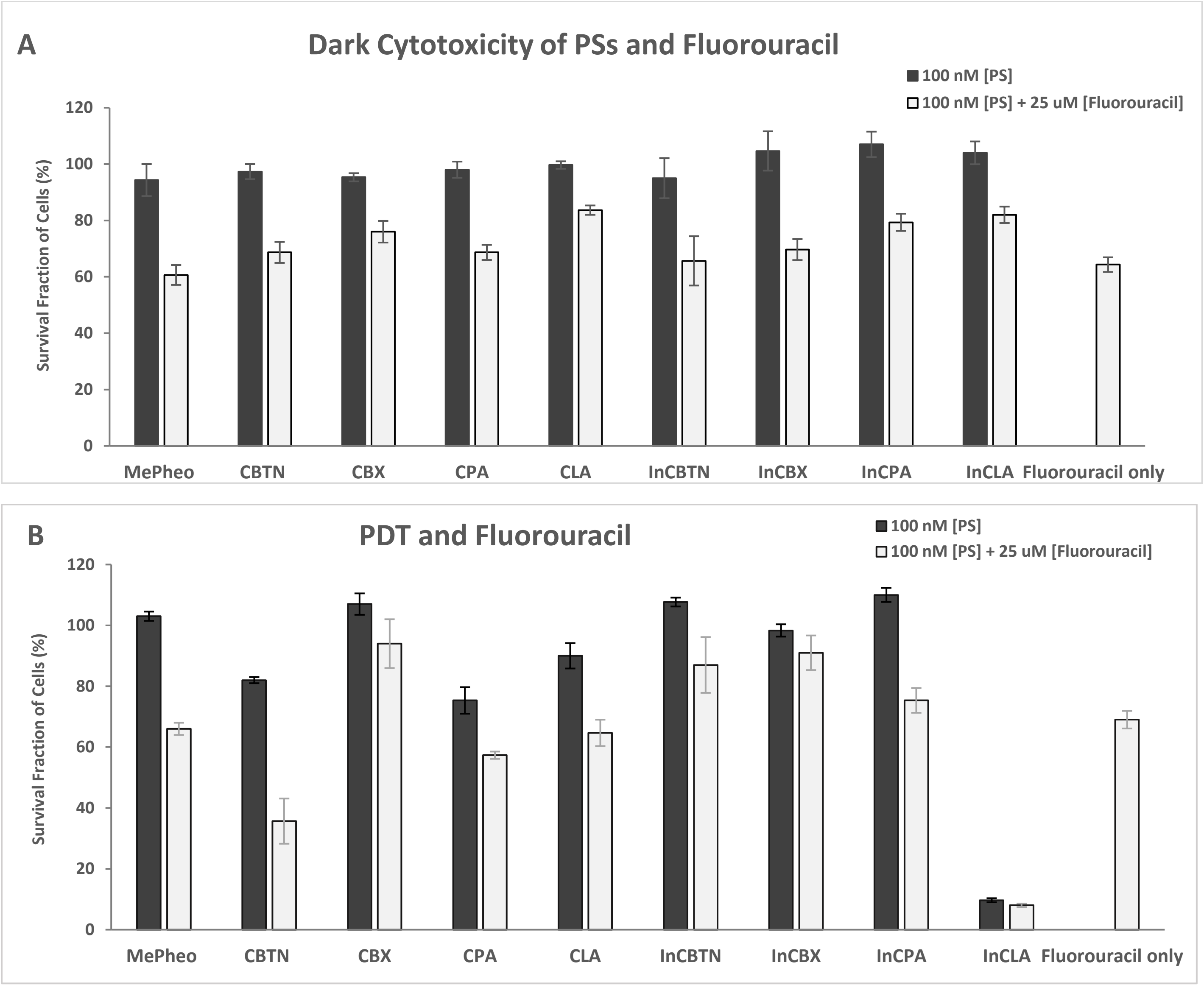

Figure S2. Cell survival assay of TNBC cells treated with photosensitizers at 100 nM and at 100 nM co-treated with 25 μM fluorouracil in the dark (A), in the presence of light (B), and compared to fluorouracil-only treated cells.

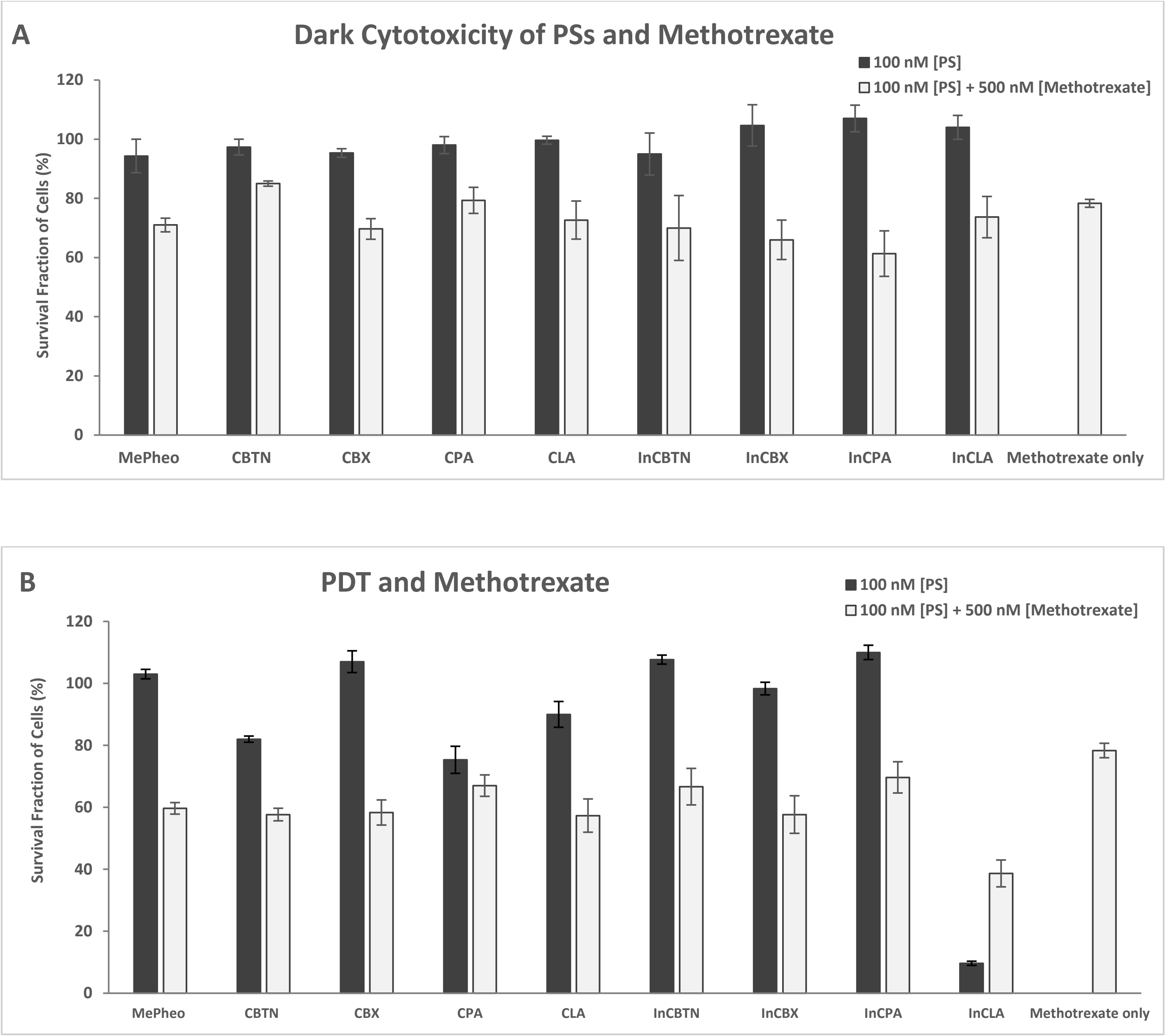

Figure S3. Cell survival assay of TNBC cells treated with photosensitizers at 100 nM and at 100 nM co-treated with 500 nM methotrexate in the dark (A), in the presence of light (B), and compared to methotrexate-only treated cells.
